# Prolonged Minocycline Treatment Impairs Motor Neuronal Survival and Glial Function in Organotypic Rat Spinal Cord Cultures

**DOI:** 10.1371/journal.pone.0073422

**Published:** 2013-08-13

**Authors:** Josephine Pinkernelle, Hisham Fansa, Uwe Ebmeyer, Gerburg Keilhoff

**Affiliations:** 1 Institute of Biochemistry and Cell Biology, Otto-von-Guericke University Magdeburg, Magdeburg, Germany; 2 Department of Plastic, Reconstructive and Aesthetic Surgery, Hand Surgery; Klinikum Bielefeld, Bielefeld, Germany; 3 Clinic of Anesthesiology, Otto-von-Guericke University Magdeburg, Magdeburg, Germany; University of Arizona, United States of America

## Abstract

**Background:**

Minocycline, a second-generation tetracycline antibiotic, exhibits anti-inflammatory and neuroprotective effects in various experimental models of neurological diseases, such as stroke, Alzheimer’s disease, amyotrophic lateral sclerosis and spinal cord injury. However, conflicting results have prompted a debate regarding the beneficial effects of minocycline.

**Methods:**

In this study, we analyzed minocycline treatment in organotypic spinal cord cultures of neonatal rats as a model of motor neuron survival and regeneration after injury. Minocycline was administered in 2 different concentrations (10 and 100 µM) at various time points in culture and fixed after 1 week.

**Results:**

Prolonged minocycline administration decreased the survival of motor neurons in the organotypic cultures. This effect was strongly enhanced with higher concentrations of minocycline. High concentrations of minocycline reduced the number of DAPI-positive cell nuclei in organotypic cultures and simultaneously inhibited microglial activation. Astrocytes, which covered the surface of the control organotypic cultures, revealed a peripheral distribution after early minocycline treatment. Thus, we further analyzed the effects of 100 µM minocycline on the viability and migration ability of dispersed primary glial cell cultures. We found that minocycline reduced cell viability, delayed wound closure in a scratch migration assay and increased connexin 43 protein levels in these cultures.

**Conclusions:**

The administration of high doses of minocycline was deleterious for motor neuron survival. In addition, it inhibited microglial activation and impaired glial viability and migration. These data suggest that especially high doses of minocycline might have undesired affects in treatment of spinal cord injury. Further experiments are required to determine the conditions for the safe clinical administration of minocycline in spinal cord injured patients.

## Introduction

The repair and regeneration of motor neurons in the injured spinal cord is clinically relevant. Depending on the severity and location of the spinal cord injury, patients may suffer from incomplete or complete motor and sensory loss of function, such as paralysis of the extremities and loss of bowel and bladder functional control [1]. Moreover, there are traumatic and non-traumatic causes of spinal cord injury, including neurodegenerative diseases. Spinal cord injury is characterized by an acute and secondary damage phase. The acute phase includes the disruption of cells and their contacts, tissue swelling, interruption of blood vessels, breakdown of the blood–brain barrier and neutrophil infiltration into the parenchyma [2]. Schnell et al. [3] compared acute inflammatory responses induced by mechanical lesions in the mouse brain and spinal cord. Mechanical spinal cord lesions resulted in a higher recruitment of neutrophils and macrophages and a larger breakdown area of the blood–brain barrier. Activated microglia release proinflammatory cytokines, chemokines, nitric oxide and superoxide free radicals which facilitate ongoing cell death by generating reactive oxygen species [4–6]. Activated astroglia increase cell-type specific proteins, such as glial fibrillary acid protein (GFAP), and secrete neurotrophic factors and pro-inflammatory cytokines [7]. In the later phase of spinal cord injury, reactive astroglia form a glial scar, thereby creating a barrier for regenerating axons. Astroglial extracellular matrix molecules, such as chondroitin sulfate proteoglycans, inhibit axonal growth beyond the scar, thereby preventing regeneration [8]. However, beneficial functions derive from the glial scar depending on the phase of injury [9–11]. In the past, several strategies have been used to enhance axonal regeneration and functional recovery after spinal cord injury, including the reduction of inflammation, inhibiting the formation of a glial scar, degradation/blockade of inhibitory molecules to the delivery of growth factors and transplantation of cells [12–14]. Nevertheless, axonal regeneration remains challenging.

Minocycline is a second-generation semi-synthetic tetracycline that exhibits antibiotic functions. In addition, minocycline demonstrates neuroprotective and anti-inflammatory actions in humans [15–17]. In rat models, minocycline reduced lesion size and apoptotic pattern after mild contusion injury of the spinal cord [18] and facilitated recovery of motor function and attenuation of mechanical hyperalgesia after a spinal cord hemisection [19]. Similar results have also been obtained in mice with an extradural compression of the spinal cord [20].

The mechanisms of minocycline action appear to result from microglial inhibition and anti-apoptotic functions. Microglial inhibition results in, among others, the downregulation of MHC II expression [21], the inhibition of the p38 MAPK pathway [22,23], a decrease in cell motility, a reduction in β-integrin and Kv1.3 potassium channel expression [24], a reduction in prostaglandin E synthase expression [25] or a reduction in the level of matrix metalloproteinases [26]. Anti-apoptotic mechanisms include a decrease in the mitochondrial permeability transition probability [27], a decrease in Ca^2+^ uptake under stress conditions [28], a reduction in reactive species formation [28,29] and the inhibition of the p38 MAPK pathway [30,31]. However, the beneficial role of minocycline has been doubted within the last few years. Several studies have reported a negative effect of minocycline in animal models of neurodegenerative disorders. Yang et al. [32] demonstrated that minocycline enhanced 1-m e t h y l-4-p h e n y l-1, 2, 3, 6-tetrahydropyridine (MPTP)-induced damage in dopaminergic neurons in mice. Diguet and colleagues [33] provided supporting results in a monkey and mouse model for Parkinson’s and Huntington’s disease, respectively, and found that minocycline treatment increased the loss of dopaminergic nerve endings and cell death. Moreover, minocycline enhanced the cell death of neurons and astrocytes *in vitro* under conditions of oxygen glucose deprivation and *in vivo* after cerebral ischemia, which was dose-dependent [34]. In a mouse model of amyotrophic lateral sclerosis, an enhancement in neuroinflammation and altered glial responses were found with minocycline treatment after disease onset [35]. In isolated rat liver mitochondria, minocycline triggered mitochondrial swelling and cytochrome c release [36]. In the peripheral nerve system (PNS), Wallerian degeneration, a prerequisite of regeneration, was significantly impaired [37].

Because of these contradictory results, we decided to investigate the effects of minocycline in a model of motor neuron survival and regeneration after injury. We used organotypic spinal cord cultures to model the survival of motor neurons and their neurite regeneration after traumatic injury. Thus, neonatal spinal cords were sectioned, thereby cutting the axons and resulting in a loss of target for the motor neurons. The slices were then cultured for 1 week to evaluate neuronal survival, neurite growth, microglial and astroglial activation after 10 or 100 µM minocycline treatments. In addition, we used organotypic spinal cord co-cultures with a peripheral nerve graft to reconstruct the ventral root after axotomy. These peripheral nerve grafts contain Schwann cells which produce neurotrophic factors after nerve injury and thereby contribute to the guidance of sprouting axons. Previous results of Haninec et al. [38] have indicated that motor neuronal outgrowth and survival are stimulated if a peripheral nerve graft is offered immediately after root avulsion. Thus, we determined the effects of minocycline treatment on neuronal survival, neurite growth and astroglial activation. To rule out glial effects, we used dispersed primary glia cell cultures and evaluated cytotoxicity and migratory functions after minocycline treatment.

## Material and Methods

### Animals

All of the animal studies were performed in accordance with the guidelines of the German Animal Welfare Act. This study was approved by the Animal Care and Use Committee of Saxony-Anhalt, Germany. A formal approval to conduct the described experiments was obtained from the animal subjects review board of our institution and can be provided upon request. All efforts were made to minimize the number of animals used and their suffering.

### Minocycline

Minocycline hydrochloride (Sigma, St. Louis, USA) was dissolved in sterile phosphate buffered saline (PBS) to obtain a stock solution of 5 mg/ml. This solution was then diluted with the respective media to the final concentrations of 10 and 100 µM used in the experiments. Our previous study revealed a beneficial effect of minocycline [39]. In this study, 2 concentrations were compared (5 and 50 µg/ml) with the higher one being the more effective. Minocycline hydrochloride has a molecular weight of 493.9 g/mol. Thus, 494 mg/ml corresponds to 1 M and 50 µg/ml to 100 µM. There have been studies with conflicting results (beneficial or harmful effects of minocycline), which were often performed using similar concentrations of minocycline. For example, Gieseler et al. [27] reported an anti-apoptotic mechanism using 100 µM minocycline in primary neuronal cultures, which decreased the mitochondrial permeability transition probability. In contrast, Kupsch and colleagues [36] found that the same minocycline concentration impaired normal mitochondrial function in isolated rat mitochondria.

Because previous studies conducted by our group and others used 100 µM [26,27,40,41], minocycline concentrations of 10 and 100 µM were selected for use in our experiments, which correlate with concentrations used in studies by Keilhoff et al. [39].

### Preparation of organotypic cultures

#### Organotypic spinal cord cultures

Organotypic spinal cord slices were prepared as described by Vyas and colleagues [42] with slight modifications. Neonatal rats (postnatal day 4) were decapitated, and their spinal cords were excised. The spinal roots and meninges were removed in dissection buffer (Hank’s balanced salt solution (HBSS), 3.4 mM NaHCO_3_, 10 mM 4-(2-hydroxyethyl)-1-piperazineethanesulfonic acid (HEPES), 33.3 mM D-glucose, 5.8 mM MgSO_4_, 0.03% bovine serum albumin (BSA), 1% penicillin/streptomycin (pen/strep)), and the lumbar spinal cord (approximately L1-L6) was cut into 350 µm transverse sections using a McIlwain tissue chopper (Mickle Laboratory Engineering, Gomshall, UK). Approximately 10 usable sections can be obtained from one lumbar spinal cord. 6-8 slices were used from 1 animal. The slices were grouped into control and minocycline-treated (+Mino) cultures. Thus, 3-4 slices obtained from the same animal were cultured on 1 Millicell membrane insert (Millipore, Billerica, USA) for the control and +Mino group and then placed into 6-well plates. Each well contained 1 ml of medium composed of 50% Eagle’s minimal essential medium (MEM), 25% HBSS, 25% fetal calf serum (FCS), 33.3 mM D-glucose, 1% pen/strep and 100 ng/ml glial cell line-derived neurotrophic factor (GDNF). Cultures were then incubated at 37° C in a humidified 6% CO_2_ atmosphere. The medium was changed 24 h after preparation and every 2-3 days thereafter.

Preparation of the organotypic spinal cord cultures induced a loss of motor neurons due to the proximal axotomy caused by the slicing. However, a representative population of motor neurons (approximately 60%) was maintained after 1 week in culture in the presence of GDNF [42,43]. Thus, all of our cultures received GDNF treatment independent of the experimental group to stabilize the motor neuron population.

#### Organotypic spinal cord-nerve graft co-cultures

Organotypic spinal cord slices were prepared as described previously [44] and above. To guide sprouting neurites and reconstruct the ventral root, the spinal cord slices were now co-cultured with a peripheral nerve graft. For these grafts, pieces of the ulnar and median nerves were harvested from the same animals, and 1 graft was placed opposing the ventral side of each spinal cord slice to reconstruct the ventral root [42]. Co-cultures were cultured and manipulated as described above.

### Minocycline intervention

According to the results of our previous study [39] the effects of a low concentration of minocycline (10 µM) and a high concentration (100 µM) were examined. Additionally, we chose 2 incubation times with minocycline incubation starting from day in vitro (DIV) 1 or DIV 4 until the end of cultivation with DIV 7. We analyzed the number of surviving motor neurons and the percentage stained area of anti-pan neuronal neurofilament (pan-NF) (neurofilament measurement). Moreover, we analyzed the percentage stained area and fluorescence intensity of anti-GFAP and anti-ionized calcium binding adaptor molecule 1 (IBA-1) to measure astroglial and microglial effects of minocycline. Percentage area of 4', 6-diamidino-2-phenylindole (DAPI) cell nuclei staining was used to indirectly evaluate the viability of the cultures.

In addition, we performed a time series of minocycline treatment (high dose, 100 µM) starting at different time points in culture: DIV 0 (day of preparation), DIV 1, DIV 3, DIV 4 or DIV 6. After the initial incubation with minocycline, the drug was subsequently added to the medium each time that the medium was changed until fixation (at DIV 7). The number of surviving motor neurons and the anti-pan-NF stained area between the control and +Mino group for each different incubation times were compared.

#### Immunohistochemical staining of organotypic cultures

Cultures were fixed after 1 week of culture by replacing the medium with 4% paraformaldehyde (PFA) overnight. For immunohistochemistry, the membranes of the Millicell inserts were separated from the carrier, and the cultures attached to the membranes were stained free-floating. Slices were washed 3 times with PBS, and nonspecific binding sites were blocked with 10% FCS and 0.3% Triton-X in PBS for 1 h. Cultures were incubated overnight with primary antibodies diluted in 10% FCS, 0.3% Triton and 0.1% NaN_3_ in PBS. The following primary antibodies were used: mouse monoclonal anti-pan-NF (1:1000, Sternberger Monoclonals, Baltimore, USA) to visualize neurons including motor neurons and neurites, rabbit polyclonal anti-GFAP (1:500, Progen, Heidelberg, Germany) to visualize astroglia and rabbit polyclonal anti-IBA-1 (1:1000, Wako Pure Chemicals Industries, Osaka, Japan) to visualize microglia. To validate the staining of anti-pan-NF we additionally used a mouse monoclonal anti-neuronal nuclei antibody (NeuN, 1:100, Chemicon, Billerica, USA) to stain the neuron populations. Primary antibody incubation was followed by 3 PBS washes and secondary antibody incubation for 3 h with anti-mouse Alexa 488 and anti-rabbit Alexa 546 (1:250, Invitrogen, Carlsbad, USA) diluted in 10% FCS and 0.3% Triton in PBS. After washing again with PBS, the nuclei were counterstained with DAPI for 15 min at 37° C to quantify the number of cell nuclei in the slices. The slices were then washed and embedded on glass slides with Immu-Mount (Thermo Scientific, Waltham, USA).

#### Fluorescence microscopy, quantification and statistics of organotypic cultures

Cultures were imaged with an AxioImager microscope and analyzed with the AxioVision Rel. 4.8 Imaging software by Zeiss (Jena, Germany). Images were taken with a 2.5 x objective and a resolution of 1388 x 1040 pixels (1.4 Megapixel, for 2.5 x objective these corresponds to 3605 µm x 2701 µm). The microscopic settings and the exposure time of the fluorescence channels were set on the basis of control slices and kept equal for the corresponding preparation.

**Figure 1:**
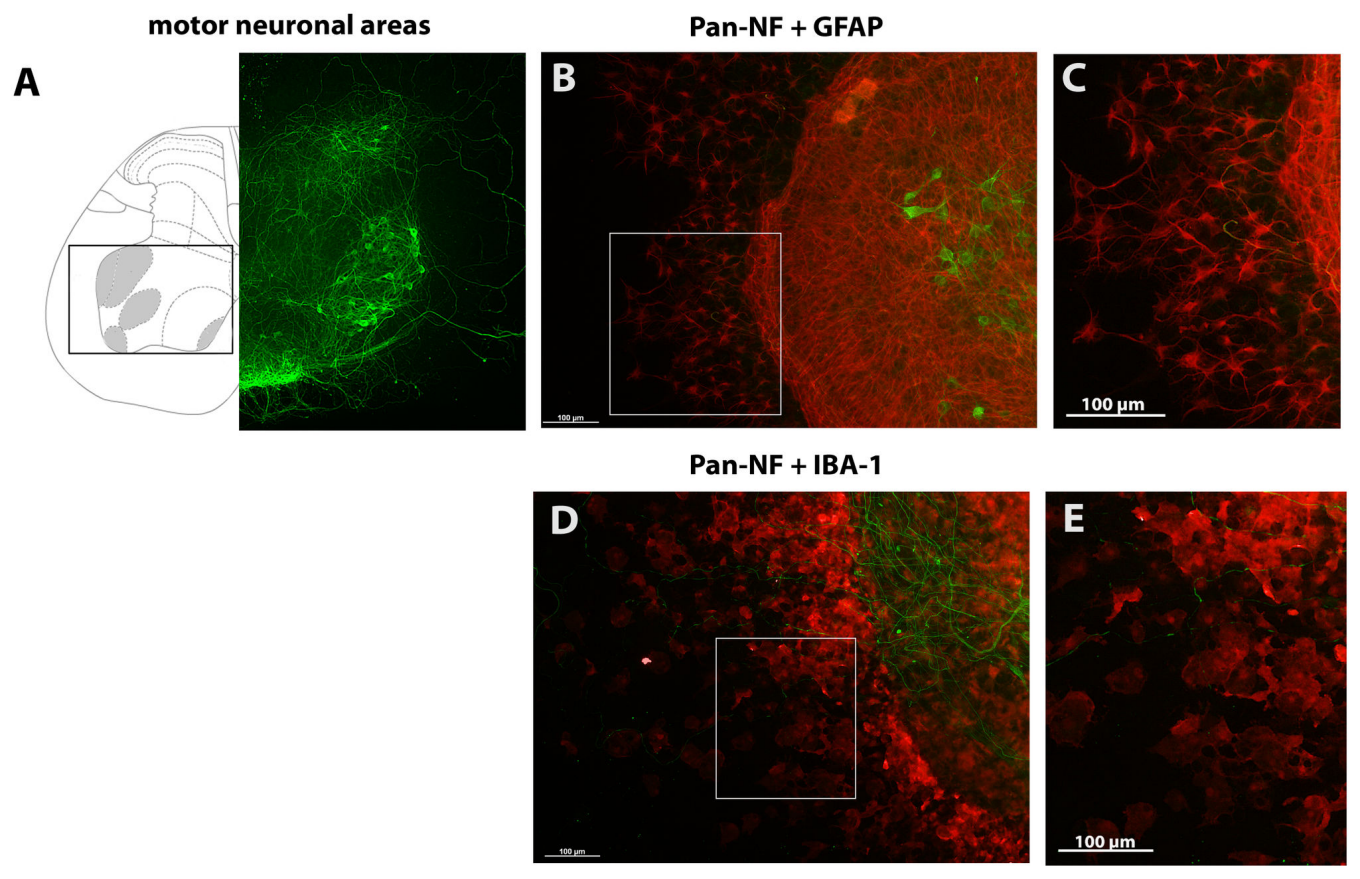
Motor neuron localization in organotypic spinal cord cultures. **(A)** Displays the anatomy of spinal cord slices at lumbar level L5 modified from [46]. Areas containing motor neurons are highlighted in grey and with a box. On the left, the spinal cord is shown as illustration; on the right side a corresponding anti-pan-NF stained spinal cord slice after 7 days of cultivation is shown. (**B** and **C**) show anti-GFAP stained astroglia in organotypic spinal cord cultures after 7 DIVs (C) magnifies stained astroglia marked with a box in (B). (**D** and **E**) illustrate anti-IBA-1 stained microglia in these cultures. (**E**) magnifies stained microglia marked with a box in (D).

Anti-pan-NF staining was used to quantify the number of surviving motor neurons. Anti-pan-NF is a general neuronal marker and is commonly used to identify neurons in tissue sections and cultures. This antibody stains not only the soma of neurons but also their neurites. One advantage of using organotypic cultures is that the tissue slices maintain their cellular organization [45]. The classification of stained neurons in our organotypic cultures was performed according to anatomical criteria [46] and cellular criteria [43]. Motor neurons were identified by their localization in the ventral horn and their large soma. A comparison of the anatomical structure, which was provided by a rat spinal cord atlas [46], and a cultured spinal cord slice after cultivation is given in Figure 1 A. Moreover, this figure includes higher magnification images for astroglia stained with anti-GFAP (Figure 1 B, C) and microglia stained with anti-IBA-1 (Figure 1 D, E). The percentage area of stained neurofilaments was additionally used to validate the results of the neuronal quantification and to quantify the neurite outgrowth. It was measured using the Auto Measure feature of the AxioVision Rel. 4.8 Imaging software. This feature was used to automatically recognize the stained area of anti-pan-NF (neurofilaments), anti-GFAP (astroglia), anti-IBA-1 (activated microglia) and DAPI (indirect slice viability) on the basis of the staining intensity. For each preparation a threshold for each channel was defined for the segmentation tool which was based on the images of control slices and kept identical for all corresponding cultures. No subtractions of background intensity were used. The Auto Measure feature calculates a percentage of recognized area and the fluorescence intensity (given in arbitrary units) above the threshold and thereby refers to the whole image area (1388 x 1040 pixels, 3605 µm x 2701 µm).

Anti-pan-NF staining was also used to verify the successful culture of organotypic cultures and co-cultures and was combined with other cell markers for immunohistochemistry. Only slices of animals which showed motor neurons in the control cultures were included in the statistical analysis. Preparations showing no stained motor neurons in the control slices were excluded along with the corresponding +Mino cultures. For each animal, up to 4 slices of control and +Mino were cultured in parallel on 1 culture insert. These replicates were analyzed individually before a mean was calculated. This mean was used as n=1 for the statistical analyses. The number of samples for each experiment is provided in the figures. In statistical analysis only corresponding controls and +Mino cultures with identical cultivation conditions and microscopic analysis were compared directly using a paired Student’s t-test.

Statistical analysis was performed using Graph Pad Prism 4 (GraphPad Software, La Jolla, USA).

### Primary dispersed glial cultures

Primary glial cultures were prepared from neonatal rats at postnatal days 2-4 [47–49]. Animals were decapitated, and their cerebral hemispheres were excised. Meninges were removed in serum-free high-glucose Dulbecco’s modified Eagle medium (DMEM) supplemented with 1% pen/strep. Cells were mechanically dissociated with 18- and 23-gauge needles and centrifuged at 1500 rpm for 5 min. The cells were resuspended in serum-supplemented high-glucose DMEM with 10% FCS and 1% pen/strep and were seeded into flasks that were pre-coated with poly-D-lysine.

Cells were incubated at 37° C in a humidified 5% CO_2_ atmosphere. The medium was changed on the second DIV to remove cell debris. After 2 weeks in culture, the culture medium was replaced with high-glucose DMEM containing 3% FCS and 1% pen/strep and used for further experiments.

The cell cultures consisted of 60.9 (± 19.0 standard deviation SD) % astroglia, 20.0 (± 12.2 SD) % microglia, 1.4 (± 3.0 SD) % neurons and 1.5 (± 1.1 SD) % oligodendroglia.

#### MTT assay to assess activity/viability of dispersed glial cell cultures

The 3-(4,5-Dimethyl-2-thiazolyl)-2,5-diphenyl-2H-tetrazolium bromide (MTT) assay measures cellular metabolic activity, thus, under defined conditions, being able to reflect cell viability and cell proliferation [50,51].

Primary glial cells were trypsinized and suspended in high-glucose DMEM containing 3% FCS and 1% pen/strep. The cells were seeded onto poly-D-lysine coated 96-well-plates with 4*10^4^ cells/well in 100 µl medium. After 24 h of cell attachment, medium only (control) or medium containing 25 µM, 50 µM, 75 µM, 100 µM and 125 µM minocycline was added to the cells. After 24 h, 48 h, 72 h and 7 days of incubation time, MTT assay was performed. The culture medium was removed and replaced with medium containing the MTT reagent at a final concentration of 1 mg/ml. The cells were incubated at 37° C in a humidified 5% CO_2_ atmosphere for 3 h. After 3 h, the supernatant was removed, and the cells were lysed with 100 µl of dimethyl sulfoxide (DMSO). The absorption was measured using a Tecan M200 microplate reader (Tecan, Männedorf, Switzerland) at 570 nm and 690 nm reference wavelengths. The mean absorption of the control cells was determined as 100%, and all other values were calculated in reference to this value. The assays were performed in triplicate (n=3 preparations) with a mean of 6 replicates each and statistically analyzed with Graph Pad Prism 4 using a 2-way-ANOVA with the factors of concentration and time, followed by a Bonferroni post-hoc test. A p-value ≤ 0.05 was considered to be statistically significant.

#### Assay to assess glial cell migration ability

A scratch assay was performed to measure the migratory ability of the cells [52]. Thus, a part of the cell layer was removed, and the closure of the gap in the cell layer was measured. To analyze the migratory function, the cells were incubated with a high minocycline concentration of 100 µM.

Primary glial cells were trypsinized and suspended in high-glucose DMEM containing 3% FCS and 1% pen/strep. The cells were seeded onto poly-D-lysine coated 6-well-plates with 6*10^5^ cells/well in 2 ml medium.

After 24 h of cell attachment, the medium was replaced with high-glucose DMEM containing 1% FCS and 1% pen/strep for 24 h to synchronize the cells. Next, the cells were treated with 100 µM minocycline and analyzed for migratory behavior. The cell monolayer was wounded after cell synchronization by removing a ^~^ 1 mm strip of cells using the sterile piston of a stepper pipette tip. A scale paper was placed underneath the well-plates and used for orientation. The cells were washed with sterile PBS to remove cell debris, and new medium containing 3% FCS without (control) or with 100 µM minocycline (+Mino) was added.

Images of the cell layer were taken immediately, 24 h, 48 h and 72 h after wounding using a Leica DMI3000 (Wetzlar, Germany). The scale on the well-plate was used to mark and locate the same position each time. Image analysis was performed using the Leica LAS AF 2.2.0 software. To analyze the leading edge, the migrating cells were outlined, and the distances were measured between the edges. The percentage values were calculated from the starting distance of the wounded cell layer.

Statistical analysis was performed using Graph Pad Prism 4, and a paired Student’s t-test with n=17 samples was used to compare the control and +Mino groups for each incubation period. A p-value ≤ 0.05 was considered to be statistically significant.

#### Western blot analysis of Connexin 43 expression in dispersed glial cell cultures

Primary glial cell cultures were incubated for 72 h with 100 µM minocycline in high-glucose DMEM containing 3% FCS and 1% pen/strep. The medium was discarded, and the cells were washed with PBS before scraping them off the culture plastic. The cells were centrifuged at 1500 rpm for 10 min, washed twice with PBS and pelleted. The cell pellets were mechanically homogenized in 20 mM Tris, 150 mM NaCl, 10% Triton-X, 10% sodium dodecyl sulphate (SDS) and CompleteMini Protease Inhibitor Cocktail (Roche, Basel, Switzerland). Proteins were separated using SDS-PAGE (5% stacking and 10% resolving gel). For gel loading, 10 µg and 2 µg protein samples were loaded after mixing with protein loading buffer (roti-Load 1, Roth, Karlsruhe, Germany). After separation, the proteins were transferred on to a nitrocellulose membrane and then washed twice with Tris buffered saline + 0.1% Tween-20 (TBS-TWEEN). The nonspecific binding sites were blocked for 2 h with TBS-TWEEN + 5% powered milk. The membranes were incubated with the following primary antibodies: anti-mouse β-actin (1 h incubation, 1:15000, Sigma Aldrich, St. Louis, USA), anti-rabbit Connexin 43 (overnight, Cx43, 1:50000, Sigma) and anti-mouse glyceraldehyde 3-phosphate dehydrogenase (overnight, GAPDH, 1:500, Chemicon) diluted in TBS-TWEEN + 5% powered milk. After washing again with TBS-TWEEN, the blots were incubated for 30 min with horseradish peroxidise-conjugated secondary antibody (anti-mouse 1:10000, anti-rabbit 1:5000, West Grove, USA) in TBS-TWEEN + 0.5% powered milk. Anti-β-actin and anti-GAPDH were developed in parallel because they differ in their molecular weight and shared the same host. The immunoreactivity was visualized with an ECL detection system (Amersham Pharmacia Biotech, Buckinghamshire, UK) and quantified with densitometry analysis using ImageJ 1.45s (National Institutes of Health, Bethesda, USA). Statistical analysis was performed with Graph Pad Prism 4 using a paired Student’s t-test with n=7 samples to compare the control and +Mino groups. A p-value ≤ 0.05 was considered to be statistically significant.

## Results

### Minocycline influenced the survival of motor neurons and the activity of glial cells in organotypic spinal cord cultures

Incubation of organotypic spinal cord slices with minocycline started at DIV 1 or DIV 4 and continued until fixation at DIV 7. For both incubation times, we compared the number of surviving motor neurons and the percentage stained area of anti-pan-NF, anti-IBA-1, anti-GFAP and DAPI between the control cultures receiving no minocycline and minocycline-treated cultures (+Mino). Fluorescence intensities were additionally measured for astroglial (anti-GFAP) and microglial (anti-IBA-1) stainings.

#### Incubation with 10 µM minocycline

**Figure 2:**
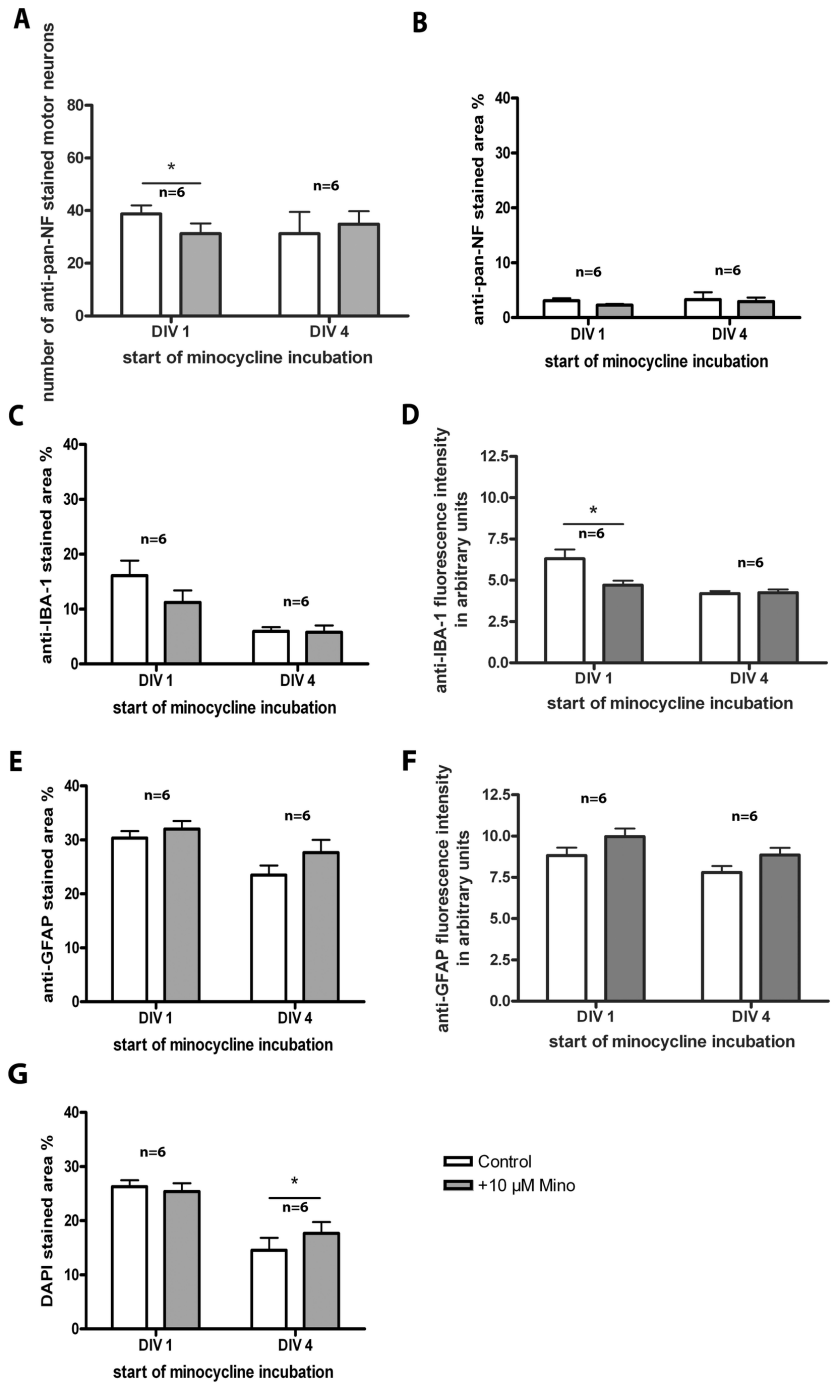
Treatment of organotypic spinal cord cultures with 10 µM minocycline. Comparison of cultures incubated from DIV 1 or DIV 4 onwards and analyzed at DIV 7. (**A**) Number of surviving anti-pan-NF stained motor neurons. Minocycline incubation starting at DIV 1, but not at DIV 4, decreased the number of surviving motor neurons significantly. (**B**) **Percentage of anti-pan-NF stained area (neurofilaments). Minocycline incubation did not alter it**. (**C**) Percentage of anti-IBA-1 stained area (microglia). Minocycline incubation did not show a significant influence on microglia pattern. (**D**)Anti-IBA-1 fluorescence staining intensity. Early (long-term) minocycline incubation reduced the fluorescence intensity (activity) of microglia significantly. (**E**) Percentage of anti-GFAP stained area (astroglia). Minocycline incubation did not show an influence on astroglia pattern. (**F**) Anti-GFAP fluorescence staining intensity. Minocycline incubation did not show an influence on the fluorescence intensity (activity) of astroglia. (**G**) Percentage of DAPI stained area. Minocycline incubation increased the percentage area of DAPI cell nuclei staining in cultures incubated with minocycline from DIV 4, but not DIV 1 onwards. For statistical analysis a paired Student’s t-test was used to compare control and +Mino for each incubation period. Number of samples n=6 for each incubation time with minocycline (1 animal = mean of up to 3 replicates). All values are means ± SD. Significant differences are marked with * and demonstrate p<0.05.

Treatment from DIV 1 onwards resulted in a significantly reduced number of surviving motor neurons (anti-pan-NF, Figure 2 A). No significant effect of early minocycline treatment on the percentage stained area of neurofilaments (anti-pan-NF, Figure 2B), microglia (anti-IBA-1, Figure 2C), astroglia (anti-GFAP, Figure 2E) and DAPI-stained cell nuclei (Figure 2G) was found. Microglia activity, demonstrated by fluorescence intensity, was significantly reduced by early minocycline treatment (Figure 2D), whereby astroglia activity was unchanged (Figure 2F).

**Figure 3:**
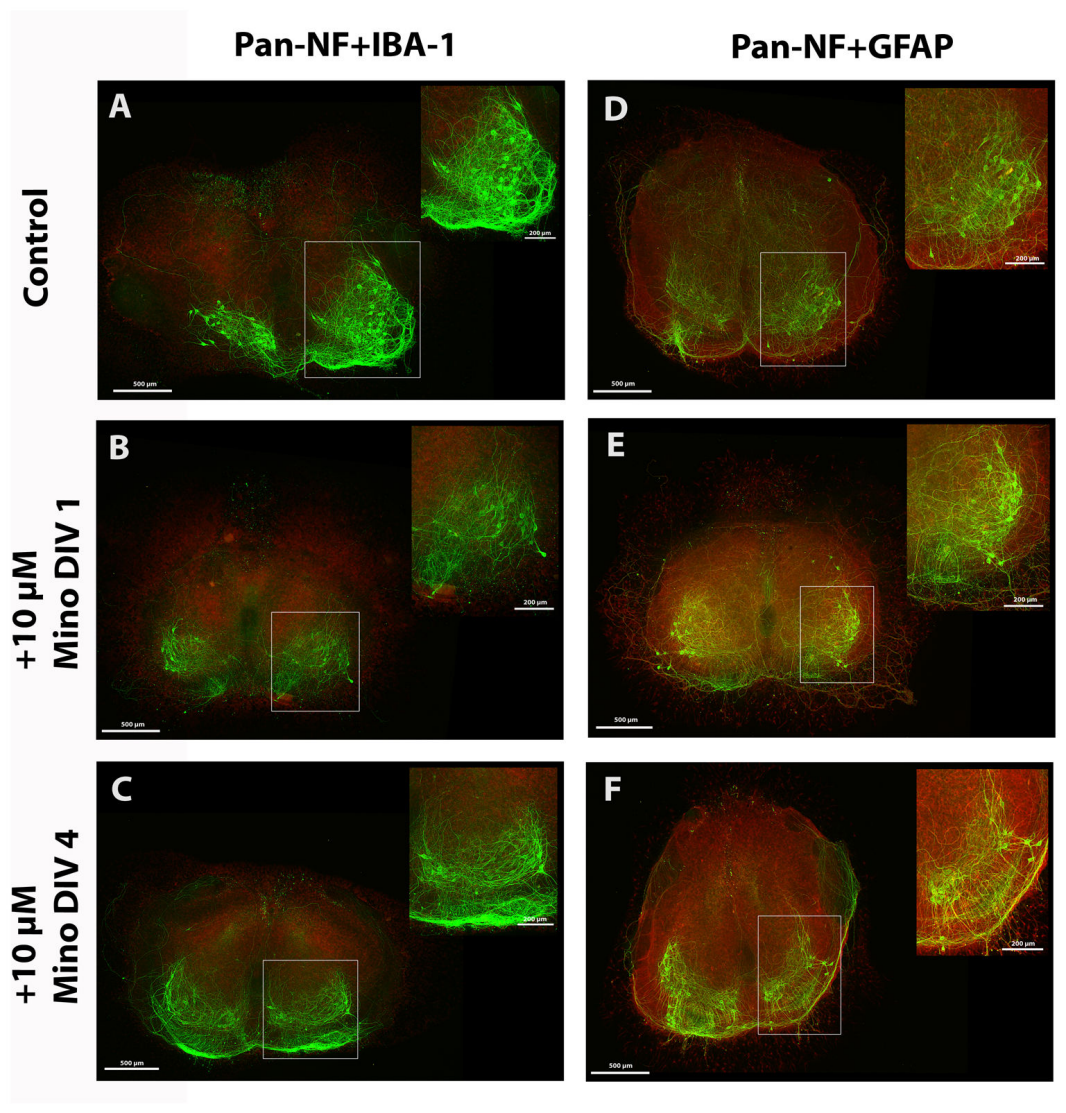
Fluorescence images of spinal cord cultures treated with 10 µM minocycline. Fluorescent images of spinal cord cultures of control (A, D) and of slices incubated with 10 µM minocycline from DIV 1 (B, E) and from DIV 4 (C, F) onwards. Slices were co-stained for anti-pan-NF (green) and anti-IBA-1 (red) in (A, B, C) or for anti-pan-NF (green) and anti-GFAP (red) in (D, E, F) at DIV 7. Motor neuronal areas are magnified and marked with a box. Control cultures show various anti-pan-NF stained motor neurons, neurons, modest microglia activation (A) and formation of a glia cover (B). All cultures treated with minocycline show control-like staining patterns. No clear differences in the staining pattern are visible. Bars = 500 µm, bars in magnified area = 200 µm.

Minocycline treatment from DIV 4 onwards did not significantly alter the number of surviving motor neurons (Figure 2 A). No significant effect of minocycline treatment on the percentage stained area of neurofilaments (anti-pan-NF, Figure 2 B), microglia (anti-IBA-1, Figure 2 C) and astroglia (anti-GFAP, Figure 2 E) as well as on the fluorescence intensities (activities) of microglia and astroglia (Figure 2 D, F) was observed. Detailed values are given in Table 1. The corresponding images of the stained cultures are shown in Figure 3 (DAPI staining not shown). A, B and C show the organotypic spinal cord cultures co-stained for anti-pan-NF and anti-IBA-1. D, E and F demonstrate the organotypic spinal cord cultures co-stained for anti-pan-NF and anti-GFAP. Control slices in Figure 3 A and D show various motor neurons and neurons with growing neurites, moderate microglia activation and an intact astroglia layer in the cultures. Slices incubated with 10 µM minocycline starting at DIV 1 (Figure 3 B and E) and DIV 4 (Figure 3 C and F) do not clearly show visible differences in staining pattern compared to the control slices.

**Table 1:** Results of organotypic spinal cord slices incubated with 10 µM minocycline in comparison with control slices.

**DIV**	**number of anti-pan-NF stained motor neurons**	**anti-pan-NF stained area in %**	**anti-IBA-1 stained area in %**	**anti-IBA-1 fluorescence intensity in arbitrary units**	**anti-GFAP stained area in %**	**anti-GFAP fluorescence intensity in arbitrary units**	**DAPI stained area in %**
**1**	31.2 ± 9.6 vs. 38.8 ± 7.9*,ratio 0.8 ± 0.2, n=6, p=0.0459	2.3 ± 0.4 vs. 3.1 ± 1.0, ratio 0.8 ± 0.3, n=6, p=0.1701	11.2 ± 5.4 vs. 16.1 ± 6.7, ratio 0.8 ± 0.4, n=6, p=0.1587	4.7 ± 0.6 vs. 6.3 ± 1.3*,ratio 0.8 ± 0.2, n=6, p=0.0423	32.0 ± 3.7 vs. 30.4 ± 3.1, ratio 1.1 ± 0.1, n=6, p=0.2592	10.0 ± 1.2 vs. 8.8 ± 1.1, ratio 1.1 ± 0.2, n=6, p=0.0995	25.4 ± 3.8 vs. 26.3 ± 2.9, ratio 1.0 ± 0.1, n=6, p=0.2403
**4**	34.9 ± 12.1 vs. 31.2 ± 20.2, ratio 1.5 ± 0.9, n=6, p=0.5442	3.0 ± 1.8 vs. 3.3 ± 3.3, ratio 1.8 ± 1.6, n=6, p=0.7231	5.8 ± 3.0 vs. 6.0 ± 1.9, ratio 1.0 ± 0.5, n=6, p=0.9044	4.3 ± 0.4 vs. 4.3 ± 0.4, ratio 1.0 ± 0.1, n=6, p=0.7735	27.7 ± 5.8 vs. 23.5 ± 4.3, ratio 1.2 ± 0.3, n=6, p=0.1184	8.9 ± 1.1 vs. 7.8 ± 0.9, ratio 1.2 ± 0.2, n=6, p=0.1283	17.7 ± 5.1 vs. 14.5 ± 5.6*,ratio 1.3 ± 0.2, n=6, p=0.0071

All values are given as mean ± SD. day in vitro (DIV), versus (vs.), number of samples (n), error level (p), * statistical significance p<0.05.

#### Incubation with 100 µM minocycline

**Figure 4:**
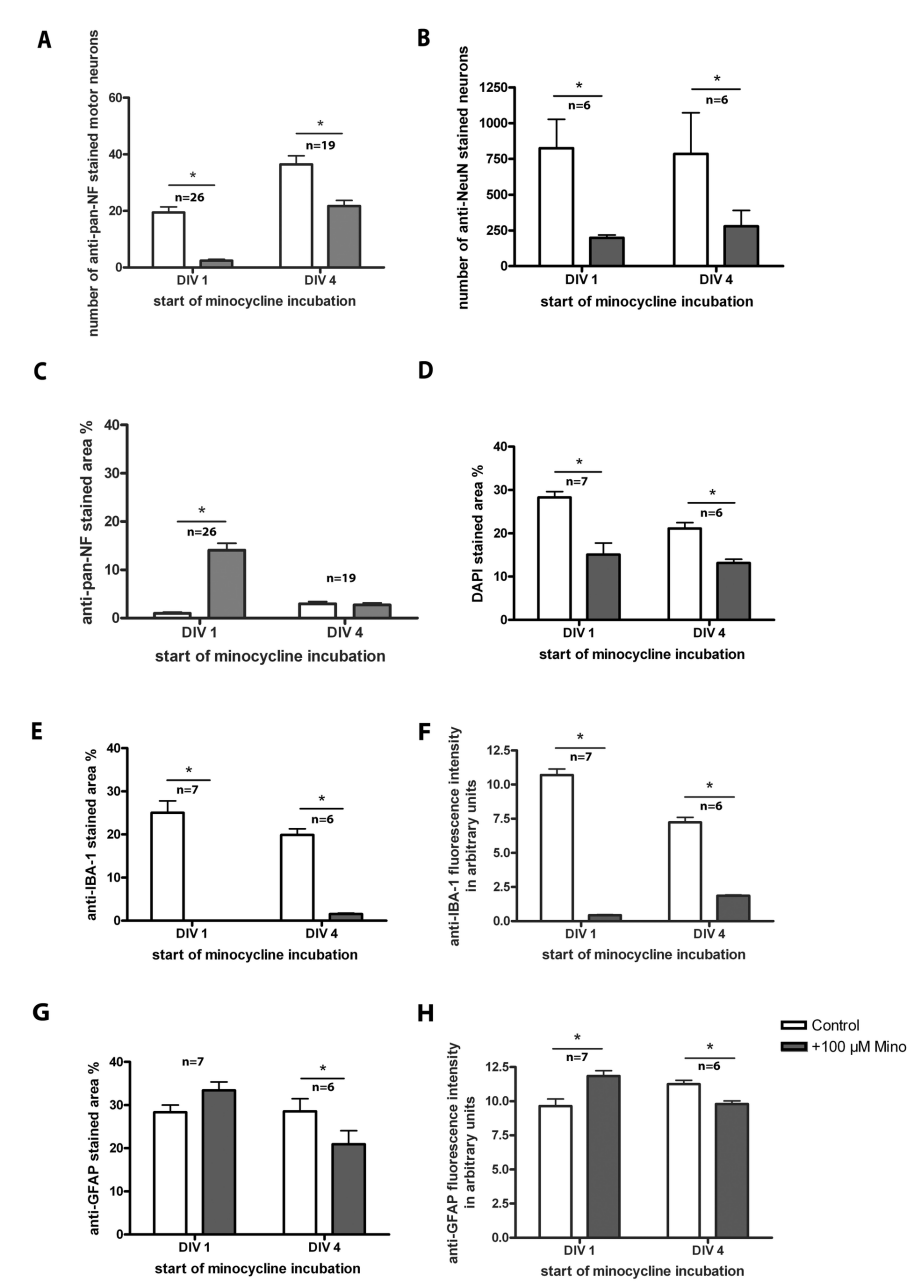
Treatment of organotypic spinal cord cultures with 100 µM minocycline. Comparison of organotypic spinal cord cultures incubated with minocycline starting at DIV 1 or DIV 4 and analyzed at DIV 7. (A) Number of surviving motor neurons (anti-pan-NF stained). Minocycline incubation decreased this number significantly compared to controls. (B) Number of anti-NeuN stained neurons. Minocycline incubation reduced this number significantly compared to controls. (C) Percentage of anti-pan-NF stained area (neurofilaments). Minocycline incubation from DIV 1 onwards increased the percentage of neurofilament staining. Minocycline incubation from DIV 4 onwards, however, did not alter it. (D) Percentage of DAPI stained area. Minocycline incubation significantly decreased the DAPI cell nuclei staining in cultures incubated from DIV 1 and DIV 4 onwards. (E) Percentage of anti-IBA-1 stained area (microglia). Minocycline incubation reduced microglia pattern significantly compared to the control. (F) Anti-IBA-1 fluorescence staining intensity. Minocycline incubation reduced the fluorescence intensity of microglia in accordance with the reduced microglia spreading. (G) Percentage of anti-GFAP stained area (astroglia). Minocycline incubation did not influence the percentage of astroglia staining significantly if cultures were incubated from DIV 1 onwards. Incubation starting at DIV 4, however, reduced the percentage of astroglia staining significantly compared to controls. (H) Anti-GFAP fluorescence staining intensity. Minocycline incubation influenced fluorescence intensity of astroglia in accordance with the changes of area. If treatment started at DIV 1, fluorescence intensity was enhanced, whereas under treatment started at DIV4 a reduction was found. For statistical analysis a paired Student’s t-test was used to compare control and +Mino for each incubation period. Numbers of samples are: number of motor neurons and percentage of anti-pan-NF area: DIV 1 n=26, DIV 4 n=19; number of anti-NeuN stained neurons n=6 (DIV 1, DIV 4); percentage stained area of anti-IBA-1, anti-GFAP and DAPI: DIV 1 n=7, DIV 4n=6 (1 animal = mean of up to 3 replicates). All values are means ± standard deviation (SD). Significant differences are marked with * and demonstrate p<0.05.

This treatment decreased the number of surviving motor neurons (determined by anti-pan-NF staining; Figure 4 A) as well as the number of neurons in general (determined by anti-NeuN staining; Figure 4 B) independently of the incubation period.

Moreover, longer (earlier starting) incubations with minocycline significantly increased the percentage of anti-pan-NF stained area (neurofilaments) of organotypic cultures compared to control slices. Incubation from DIV 4 onwards did not alter the percentage of neurofilament area (Figure 4 C).

As expected for a “microglia inhibitor”, minocycline clearly decreased the microglial-stained percentage area of the organotypic spinal cord cultures (Figure 4 E) as well as the IBA-1 fluorescence intensity (Figure 4 F) in both incubation conditions compared to control cultures. The astroglial percentage stained area (Figure 4 G) was not significantly influenced in cultures incubated with minocycline from DIV 1 onwards but significantly decreased in cultures incubated from DIV 4 onwards. The GFAP-fluorescence intensity (Figure 4 H) showed similar results, whereat the increase under long-time minocycline incubation (early start at DIV 1) is now also significant.

The DAPI-stained area percentage was reduced after minocycline treatment (Figure 4 D). DAPI-stained cell nuclei and the percentage of the stained area can be used as an indirect measurement of slice culture viability. Thus, our results indicate a reduction in the viability of the slices after incubation with minocycline.

**Figure 5:**
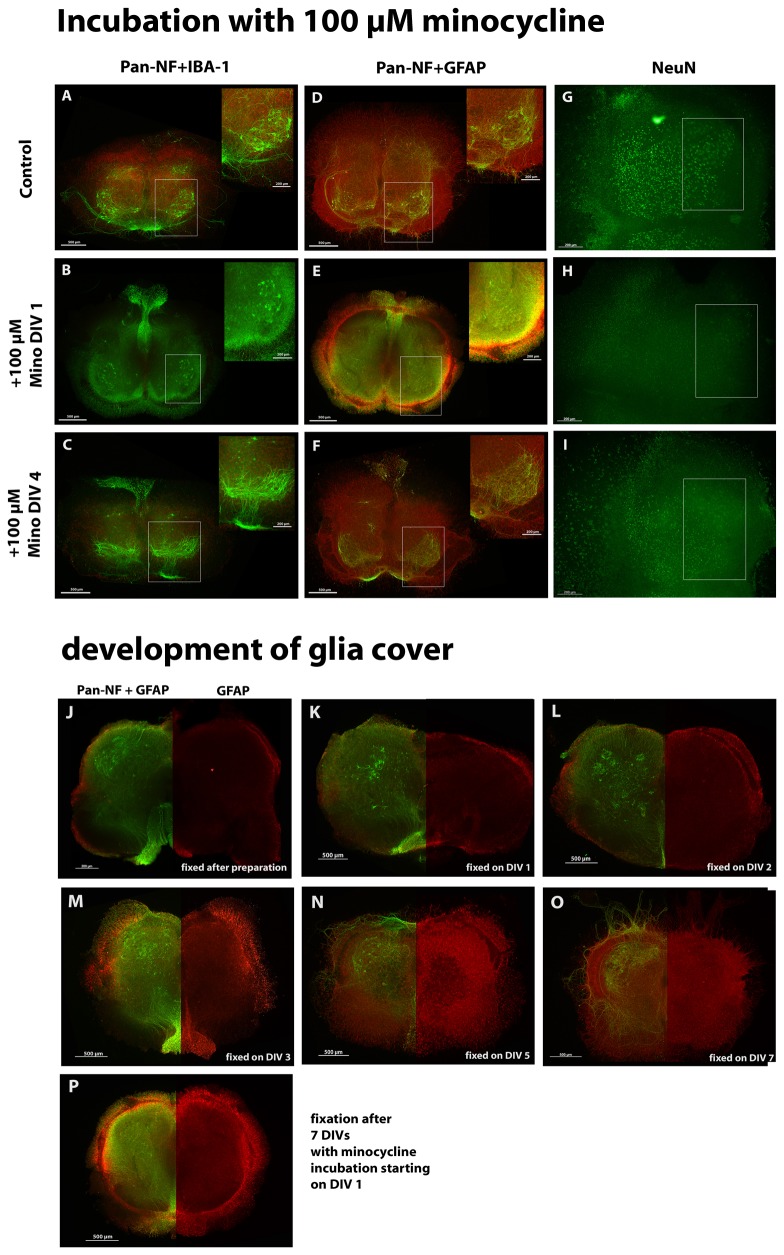
Fluorescence images of spinal cord cultures treated with 100 µM minocycline. (A, D, G) controls; (B, E, H) incubation starting at DIV 1; (C, F, I) incubation from DIV 4 onwards. Slices were co-stained for anti-pan-NF (green) and anti-IBA-1 (red) in (A, B, C), for anti-pan-NF (green) and anti-GFAP (red) in (D, E, F) or for anti-NeuN (green) in (G, H, I). Motor neuronal areas are magnified in (A–F) and marked with a box (A–I). (A) Control cultures show various anti-pan-NF stained motor neurons, neurons, modest microglia activation, (D) glia cover formation and (G) anti-NeuN stained neuron populations. Cultures treated from DIV 1 onwards displayed (B) a lack of motor neurons and microglial staining, (E) a peripheral astroglial distribution and (H) a lack of anti-NeuN stained neurons. Cultures treated from DIV 4 onwards showed (C) less motor neurons and low microglia activation, (F) the formation of a glia cover on the slice surface and (I) some anti-NeuN stained neurons. Images (J–O) illustrate the development of the glia cover during cultivation. Slices are shown double stained with anti-pan-NF and anti-GFAP on the left and anti-GFAP staining only on the right. (J) displays a slice fixed after preparation (no cultivation). A peripheral anti-GFAP staining is visible. Slices were fixed and stained after 1 DIV (K), 2 DIV (L), 3 DIV (M), 5 DIV (N) or 7 DIV (O) of cultivation. The glial distribution changes over time from a peripheral distribution to a glia cover. Bars in (A–F, J–P) = 500 µm and in magnified area = 200 µm. Bars in (G–I) = 200 µm.

All of the values can also be found in Table 2. Representative images are shown in Figure 5 (DAPI staining not shown). At DIV 7, control slices in Figure 5 showed various motor neurons and neurons with growing neurites (anti-pan-NF, Figure 5 A), moderate microglia activation (anti-IBA-1, Figure 5 A) and an intact astroglia cover (GFAP, Figure 5D). Figure 5 G displayed a high number of anti-NeuN stained neurons. Cultures incubated with minocycline starting at DIV 1 showed nearly no surviving motor neurons; however, other neurons (most likely interneurons and sensory neurons) were present (Figure 5 B, E). Anti-NeuN revealed no longer clear neuron populations (Figure 5 H). Moreover, inhibition of microglial activation was observed (anti-IBA-1; Figure 5 B). Interestingly, these cultures showed a peripheral distribution of astroglia instead of the mentioned above glia cover (anti-GFAP, Figure 5 E).

**Table 2:** Results of organotypic spinal cord slices incubated with 100 µM minocycline in comparison with control slices.

**DIV**	**number of anti-pan-NF stained motor neurons**	**number of anti-NeuN stained neurons**	**anti-IBA-1 stained area in %**	**anti-IBA-1 fluorescence intensity in arbitrary units**	**anti-GFAP stained area in %**	**anti-GFAP fluorescence intensity in arbitrary units**	**DAPI stained area in %**
**1**	2.4 ± 2.6 vs. 19.4 ± 10.2*,ratio 0.1 ± 0.2, n=26, p<0.0001	198.6 ± 48.3 vs. 824.9 ± 493.3*,ratio 0.4 ± 0.3, n=6, p=0.023	0.0 ± 0.0 vs. 25.0 ± 7.3*,ratio 0.0 ± 0.0, n=7 p<0.001	0.4 ± 0.1 vs. 10.7 ± 1.1*,ratio 0.0 ± 0.0, n=7, p<0.0001	33.4 ± 5.2 vs. 8.4 ± 4.4, ratio 1.2 ± 0.4, n=7, p=0.18	11.9 ± 1.0 vs. 9.6 ± 1.4*,ratio 1.3 ± 0.2, n=7, p=0.0066	15.1 ± 6.5 vs. 28.3 ± 3.3*,ratio 0.5 ± 0.2, n=6, p=0.0014
**4**	21.7 ± 8.7 vs. 36.5 ± 12.9*,ratio 0.6 ± 0.2, n=19, p<0.0001	278.6 ± 269.5 vs. 785.2 ± 703.4*,ratio 0.4 ± 0.1, n=6, p=0.041	1.6 ± 0.5 vs. 19.9 ± 3.5*,ratio 0.1 ± 0.0, n=6, <0.0001	1.9 ± 0.1 vs. 7.2 ± 0.9*,ratio 0.3 ± 0.0, n=6, p<0.0001	20.9 ± 7.7 vs. 28.5 ± 7.3*,ratio 0.7 ± 0.2, n=6, p=0.0064	9.8 ± 0.6 vs. 11.3 ± 0.7*,ratio 0.9 ± 0.1, n=6, p=0.0033	9.8 ± 5.2 vs. 19.2 ± 3.7*,ratio 0.5 ± 0.3, n=21, p<0.0001

All values are given as mean ± SD. day in vitro (DIV), versus (vs.), number of samples (n), error level (p), * statistical significance p<0.05.

Cultures with shorter/later minocycline incubation (starting at DIV 4) also demonstrated a loss of anti-pan-NF stained motor neurons (Figure 5 C, F) and anti-NeuN stained neurons (Figure 5 I) but to a lesser extend as in long/early-treated cultures. Moreover, in these cultures the formation of the astroglia cover (GFAP) seemed to be intact (Figure 5 F).

The covering of the organotypic slices with astroglia is a feature of membrane-based cultures with one surface having air contact [53]. A typical regime of astroglial covering is shown in the second part of Figure 5. Freshly prepared untreated control spinal cord slices showed a peripheral distribution of GFAP staining (Figure 5J). During cultivation, the glia started to cover the slices until they formed a complete coverage as it is seen in the time series (Figure 5 K–O). As demonstrated in Figure 5 E and P, the formation of such astroglia cover was hampered by early/long-term incubation with high-dosed minocycline. Because the coverage of slices is based on a migration of astroglia, we developed the idea that minocycline might influence the glial migration.

**Figure 6:**
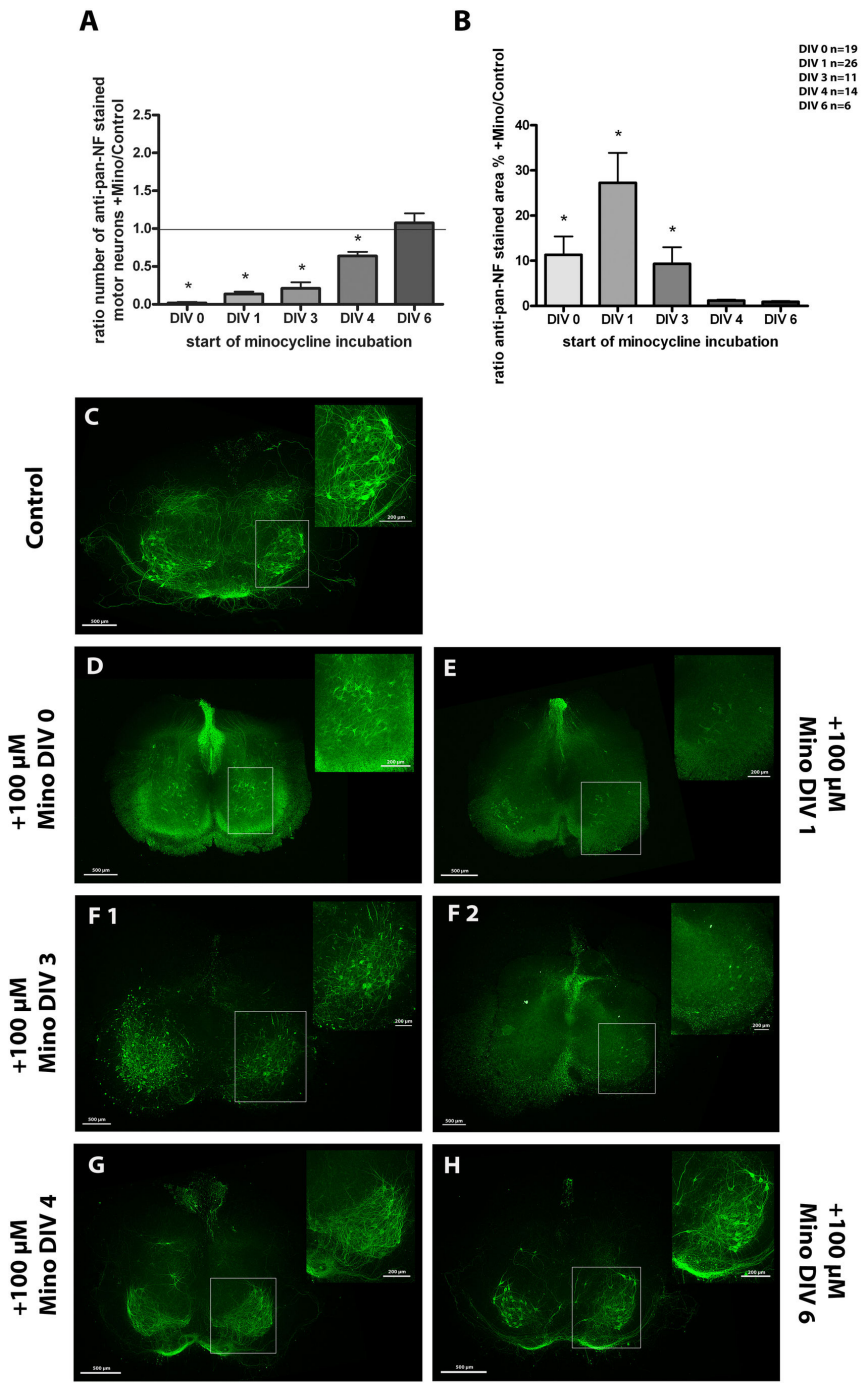
Time series of organotypic spinal cord cultures treated with 100 µM minocycline. (A) shows the number of motor neurons for cultures incubated starting at DIV 0, 1, 3, 4 or 6. In result of minocycline incubation, the number of motor neurons was reduced whereby earlier (and thus longer) incubation times were more effective. One-day treatment (starting at DIV 6) did not influence the number of motor neurons compared to controls. Values show the ratio of +Mino/controls mean ± SD. Ratio of 1 is marked as line to illustrate +Mino value = control value. (B) demonstrates the anti-pan-NF percentage stained neurofilament area for the various incubation times. Minocycline supplement from DIV 0, 1 and 3 onwards resulted in an increased stained area with DIV 1 showing the highest value. In cultures from DIV 4 and 6 onwards, the percentage stained area did not change compared to the control. Statistical analysis was done with a Student’s t-test to compare control and +Mino for each incubation time. Number of samples was n=19 for DIV 0, n=26 for DIV 1, n=11 for DIV 3, n=14 for DIV 4 and n=6 for DIV 6 (1 animal = mean of up to 3 replicates). Significant differences compared to the control are marked with * and demonstrate p<0.05. (C-H) Fluorescence images of organotypic spinal cord cultures stained for anti-pan-NF in green to visualize neurons, especially motor neurons and neuronal filaments. Motor neuronal areas are magnified and marked with a box. Slices were cultivated for 7 days and incubated from DIV 0, 1, 3, 4 or 6 onwards with minocycline. (C) control culture showing various regular motor neurons, neurons and neurites; (D) culture incubated from DIV 0 onwards lacking motor neurons but displaying a clear staining of other neurons like interneurons and sensory neurons. (E) culture incubated from DIV 1 onwards demonstrating only a few neurons at all; (F 1 and 2) cultures incubated from DIV 3 onwards showed degenerating motor neurons like it is seen in (F 1) or lacked motor neurons like it is seen in (F 2). (G) culture incubated from DIV 4 onwards showing regular motor neurons and neurites (H) culture incubated from DIV 6 onwards showing motor neurons and their neurites. Bars = 500 µm, bars in magnified area = 200 µm.

Furthermore, we performed a time series study at a high minocycline concentration to analyze the effects on motor neurons in more detail. Here, +Mino cultures were incubated with minocycline for different periods (starting at DIV 0, DIV 1, DIV 3, DIV 4 or DIV 6). The results are demonstrated in Figure 6. The number of motor neurons was dramatically decreased if the cultures were incubated with minocycline for the entire cultivation period of 7 days (from DIV 0 onwards) compared to control cultures. With shorter/later incubation periods the number of surviving motor neurons increased reaching control levels in cultures incubated with minocycline starting at DIV 6 (Figure 6 A).

The comparison of the anti-pan-NF-stained areas among the different incubation times is shown in Figure 6 B. Significantly higher levels of staining were revealed in the cultures incubated from DIV 0, 1 and 3 onwards if compared to controls with being highest in the cultures incubated with minocycline starting at DIV 1. Minocycline treatment from DIV 4 or 6 onwards showed no significant differences in the neurofilament-stained area compared to controls. Detailed values can be found in Table 3.

**Table 3:** Timeline results of organotypic spinal cord slices incubated with 100 µM minocycline in comparison with control slices.

**DIV**	**number of anti-pan-NF stained motor neurons**	**anti-pan-NF stained area in %**
**0**	0.3 ± 0.8 vs. 27.8 ± 15.2*,ratio 0.0 ± 0.1, n=19, p<0.0001	9.9 ± 5.8 vs. 2.3 ± 1.5*,ratio 11.3 ± 17.7, n=19, p<0.0001
**1**	2.4 ± 2.6 vs. 19.4 ± 10.2*,ratio 0.1 ± 0.2, n=26, p<0.0001	14.1 ± 7.1 vs. 1.0 ± 1.1*,ratio 27.3 ± 33.7, n=26, p<0.0001
**3**	3.6 ± 5.9 vs. 19.5 ± 14.6*,ratio 0.2 ± 0.3, n=11, p=0.0067	8.2 ± 3.3 vs. 2.9 ± 2.8*,ratio 9.3 ± 12.4, n=11, p=0.0002
**4**	21.7 ± 8.7 vs. 36.5 ± 12.9*,ratio 0.6 ± 0.2, n=19, p=0.0001	2.5 ± 1.5 vs. 2.7 ± 1.6, ratio 1.2 ± 0.9, n=19, p=0.58
**6**	47.1 ± 15.1 vs. 47.8 ± 21.3, ratio 1.1 ± 0.3, n=6, p=0.94	1.7 ± 1.1 vs. 2.0 ± 1.1, ratio 0.9 ± 0.5, n=6, p=0.44

All values are given as mean ± SD. day in vitro (DIV), versus (vs.), number of samples (n), error level (p), * statistical significance p<0.05.

Respective representative fluorescent images are also shown in Figure 6. The control cultures (Figure 6 C) demonstrated regular motor neurons, neurons and neurites. Moreover, the tissue organization was conserved during culture. In contrast, the cultures incubated with minocycline for the entire cultivation period of 7 days (from DIV 0 onwards) lacked motor neurons but displayed a clear staining of other neurons such as interneurons and sensory neurons (Figure 6 D). Cultures incubated from DIV 1 onwards demonstrated only a few neurons, if at all (Figure 6 E). This difference in neuronal survival could be due to the stabilization of the organotypic cultures after preparation. DIV 0 +Mino cultures received minocycline directly after preparation whereas the DIV 1 +Mino cultures were allowed to stabilize one day in the incubator before drug administration.

Moreover, cultures incubated from DIV 3 onwards revealed two different features: degenerating motor neurons (Figure 6 F1) or the absence of motor neurons (Figure 6 F2). Minocycline treatment from DIV 4 and DIV 6 onwards displayed various regular motor neurons, neurons and neurites without any clear visible differences in the staining pattern to the control cultures (Figure 6 G, H).

### Minocycline influenced the survival of motor neurons and the activity of glial cells in organotypic spinal cord-nerve graft co-cultures

In addition to the spinal cord cultures, we used spinal cord co-cultures with a peripheral nerve graft to reconstruct the ventral root. Motor neuron cell death is highest with a proximal injury of their axons; however, this cell death can be delayed by the placement of grafts, which can bridge the gap [54,55]. Thus, a peripheral nerve graft in this co-culture model served 2 functions: first, it increased motor neuronal survival because it offers a scaffold for motor neurons to grow in, and second, it provides a source of Schwann cells, which have been shown to promote axonal regeneration in spinal cord injury [56–58].

#### Incubation with 100 µM minocycline

**Figure 7:**
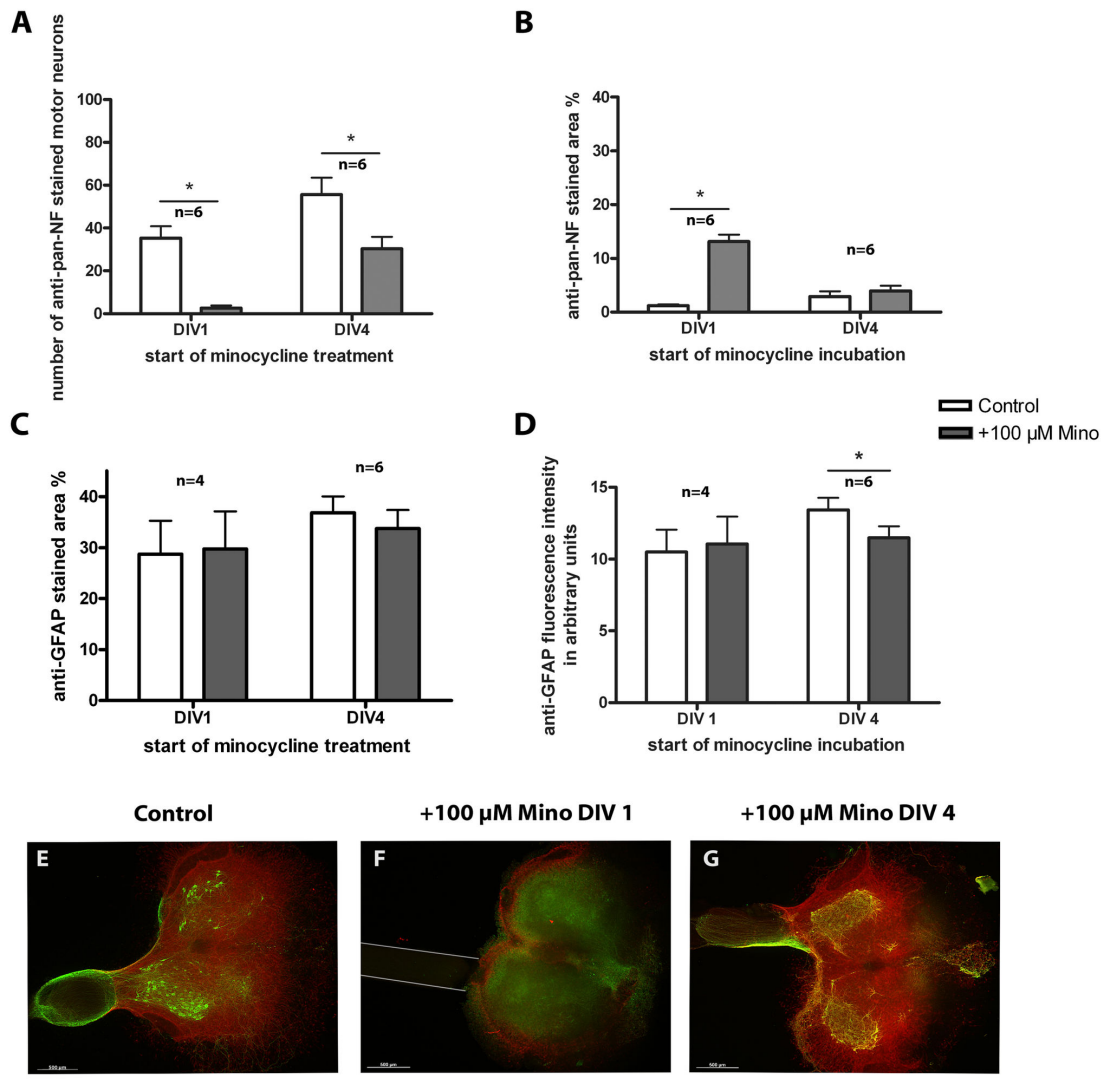
Treatment of organotypic spinal cord co-cultures with 100 µM minocycline. Comparison of control and +Mino organotypic spinal cord co-cultures cultured for 7 days. +Mino co-cultures were incubated starting at DIV 1 or DIV 4. (A) number of motor neurons. Minocycline reduced the number of motor neurons compared to the control. (B) anti-pan-NF percentage stained area. Minocycline incubation from DIV 1 onwards resulted in an increased stained neurofilament respectively neurites area compared to the control. Incubation from DIV 4 onwards did not affect the percentage stained area. (C) anti-GFAP percentage stained area. (D) anti-GFAP fluorescence intensity. Neither astroglial percentage stained area nor fluorescence intensity was altered by minocycline. To compare controls and +Mino a paired Student’s t-test was used. Number of samples was n=6 except DIV 1 DAPI stained area with n=4 (1 animal = mean of up to 3 replicates). All values are means ± SD. Significant differences are marked with * and demonstrate p<0.05. (E–G) Fluorescence images of organotypic spinal cord co-cultures. A control culture is illustrated in (E) showing anti-pan-NF (green) stained motor neurons which grew neurites into the reconstructed ventral root. Astroglia (anti-GFAP, red) formed a glia cover on the surface of the slice. (F) demonstrates a co-culture incubated with minocycline from DIV 1 onwards lacking motor neurons and fiber outgrowth to the peripheral nerve graft that was opposed to the ventral side of the spinal cord slice (marked with grey **lines**). Astroglia staining shows peripheral distribution. (G) shows a co-culture treated from DIV 4 onwards. This culture shows several motor neurons, reconstruction of the ventral root and a glia cover. Bars = 500 µm.

Treatment starting at DIV 1 resulted in similar effects (Figure 7, Table 4) as observed in the organotypic spinal cord cultures without a peripheral nerve graft. The number of motor neurons was reduced and the percentage of anti-pan-NF stained area was increased (Figure 7 A, B), both to the same extent as in the respective organotypic cultures. Starting at DIV 4, minocycline treatment also led to a reduction in the number of motor neurons but did not affect the stained area percentage of anti-pan-NF (neurofilaments and neurites, Figure 7 A, B). Detailed values can be found in Table 4. Thus, nerve grafting was not able to rescue minocycline-induced motor neuron cell death. Moreover, minocycline treatment had no effect on the percentage of anti-GFAP-stained area (Figure 7 C) or GFAP fluorescence intensity (Figure 7 D) but influenced the astroglial pattern in the same manner as observed in organotypic spinal cord slices.

**Table 4:** Results of organotypic spinal cord co-cultures with reconstructed ventral root incubated with 100 µM minocycline in comparison with control slices.

**DIV**	**number of anti-pan-NF stained motor neurons**	**anti-pan-NF stained area in %**	**anti-GFAP stained area in %**	**anti-GFAP fluorescence intensity in arbitrary units**
**1**	2.6 ± 3.1 vs. 35.3 ± 13.6*,ratio 0.1 ± 0.1, n=6, p=0.0016	13.1 ± 3.2 vs. 1.2 ± 0.7*,ratio 12.7 ± 4.5, n=6, p=0.0001	29.7 ± 14.8 vs. 28.7 ± 13.1, ratio 1.0 ± 0.1, n=4, p=0.45	11.1 ± 3.8 vs. 10.5 ± 3.1, ratio 1.0 ± 0.1, n=4, p=0.2531
**4**	30.3 ± 13.6 vs. 55.6 ± 19.5*,ratio 0.6 ± 0.2, n=6, p=0.0063	3.9 ± 2.4 vs. 2.9 ± 2.3, ratio 1.9 ± 1.8, n=6, p=0.26	33.7 ± 8.9 vs. 36.8 ± 7.9, ratio 0.9 ± 0.1, n=6, p=0.06	11.5 ± 2.0 vs. 13.4 ± 2.1*,ratio 0.9 ± 0.0, n=6, p<0.0001

All values are given as mean ± SD. day in vitro (DIV), versus (vs.), number of samples (n), error level (p), * statistical significance p<0.05.

Representative fluorescent images are also shown in Figure 7. Control co-cultures illustrated various anti-pan-NF-stained motor neurons extending their neurites into the reconstructed ventral root (Figure 7 E). In addition, astroglia formed a glia cover on the surface of the slice. Minocycline incubation from DIV 1 onwards resulted in motor neuron death and subsequently in reduced fiber outgrowth into the nerve graft (Figure 7 F). Astroglial staining revealed a peripheral distribution (not a cover), as observed in organotypic spinal cord cultures in the absence of a peripheral nerve graft. In Figure 7 G, a co-culture treated with minocycline from DIV 4 onwards is shown. This culture exhibited several motor neurons and other neurons, a reconstruction of the ventral root by growing motor neuron neurites and an intact glia cover.

### Minocycline influenced the functional activity of primary glial cultures

Because high-dosed minocycline obviously influenced the astroglia in organotypic cultures we aimed to analyze this phenomenon in greater detail. Therefore, dispersed primary glial cell cultures were used in subsequent experiments.

#### MTT assay

**Figure 8:**
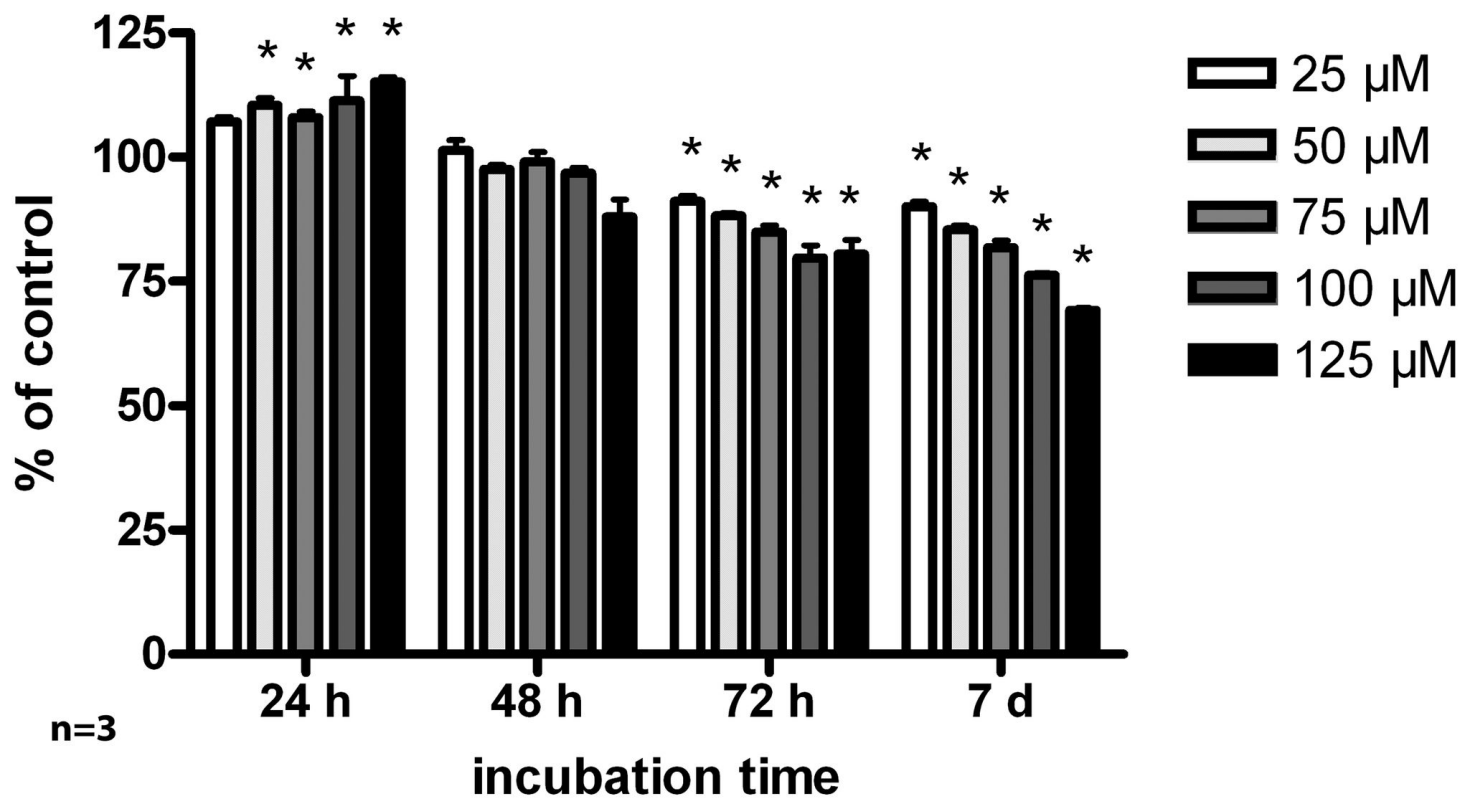
Cytotoxicity of minocycline. MTT assay of glial cell cultures incubated with increasing minocycline concentrations (25, 50, 75, 100 and 125 µM) and incubation times (24, 48, 72 h and 7 days). Cytotoxicity of minocycline increased with incubation time and concentration. Statistical analysis was done with a 2-way ANOVA. Significant differences to the control are marked with * and demonstrate p<0.05. The assay was done in triples (n = 3 preparations) with a mean of 6 replicates each.

Incubation of primary glial cultures with increasing concentrations of minocycline revealed time- and concentration-dependent effects (Figure 8). Detailed values can be found in Table 5. 24 h incubation with 50 µM and higher concentrations of minocycline resulted in increased cell metabolic activity/viability. 48 h minocycline incubation, however, decreased the levels of cell metabolic activity/viability back to control levels. Toxic effects of minocycline were found after 72 h of incubation for all minocycline concentrations used compared to control cells. After 7 days, this effect was more evident.

**Table 5:** Results of MTT assay with primary glial cell cultures which were incubated at different times with increasing minocycline concentrations.

**incubation time**	**25 µM Mino cell activity/viability in % of control**	**50 µM Mino cell activity/viability in % of control**	**75 µM Mino cell activity/viability in % of control**	**100 µM Mino cell activity/viability in % of control**	**125 µM Mino cell activity/viability in % of control**
**24 h**	107.1 ± 1.7, n=3, p>0.05	110.5 ± 2.5*,n=3, p<0.01	107.9 ± 2.3*,n=3, p<0.05	111.3 ± 8.8*,n=3, p<0.001	115.2 ± 1.6*,n=3, p<0.001
**48 h**	101.3 ± 3.5, n=3, p>0.05	97.5 ± 1.4, n=3, p>0.05	99.1 ± 3.3, n=3, p>0.05	96.9 ± 1.8, n=3, p>0.05	87.9 ± 6.2*,n=3, p<0.001
**72 h**	91.2 ± 1.7*,n=3, p<0.05	88.2 ± 1.1*,n=3, p<0.001	85.0 ± 2.2*,n=3, p<0.001	79.8 ± 4.3*,n=3, p<0.001	80.4 ± 5.1*,n=3, p<0.001
**7 days**	90.0 ± 1.6*,n=3, p<0.01	85.3 ± 1.6*,n=3, p<0.001	81.8 ± 2.5*,n=3, p<0.001	76.3 ± 0.7*,n=3, p<0.001	69.2 ± 0.8*,n=3, p<0.001

All values are given as mean ± SD. Minocycline supplement (Mino), number of samples (n), error level (p), * statistical significance p<0.05.

#### Migration assay

**Figure 9:**
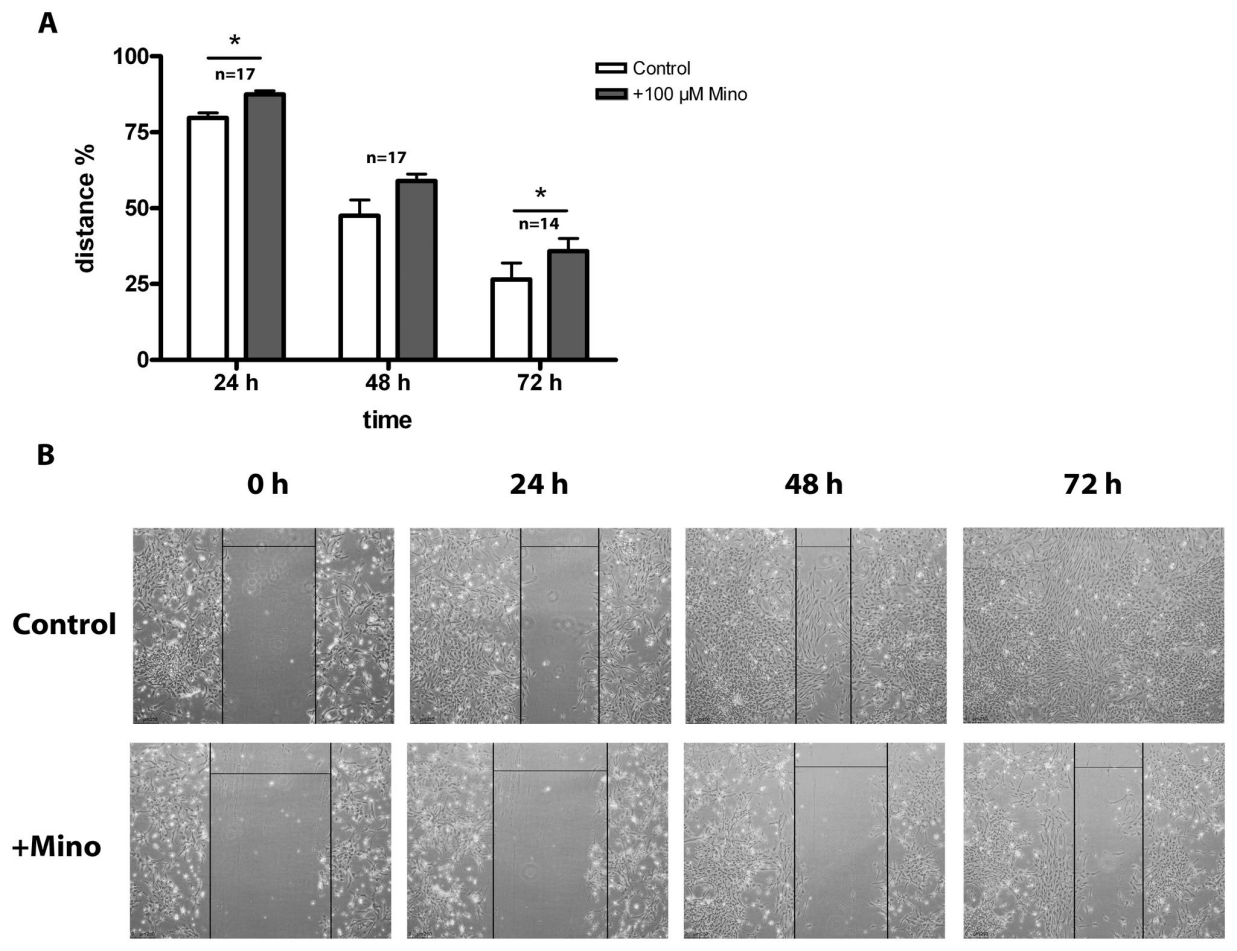
Scratching Assay of primary glial cell cultures. Cells were incubated with 100 µM minocycline for 72 h after wound scratching. All values in (A) are given as mean distance % of the starting distance of the wound ± SD. Closure of the wound was delayed in minocycline incubated cells compared to the control after 24 and 72 h. Images in (B) show phase contrast images of cultures with control cells and minocycline treated cells after 0, 24, 48 and 72 h incubation time. Wound distances are marked with black **lines**. Minocycline delayed wound closure compared to the control. Bars illustrate 250 µm. Statistical analysis was done with a paired Student’s t-test. Significant differences are marked with * and demonstrate p<0.05. The number of samples was n = 17 for 24 and 72 h and n=14 for 48 h.

Incubation with high-dosed minocycline starting at DIV 1 hampered the astroglia cover formation in organotypic cultures. To determine whether this effect was due to an impairment of glial migration behavior, we used the scratch assay. In this assay, the cell layer was wounded, and the distance between the leading edges was measured over 3 days. Once the cell layer was scratched, minocycline was added to the culture, which resulted in a lower migration rate of cells into the scratched wound area compared to control cells (Figure 9 A). Detailed values can be found in Table 6.

**Table 6:** Results of scratching assay (wound closure) with primary glial cell cultures which were incubated with 100 µM minocycline up to 72 h.

**incubation** time	**control % wound closure of starting distance**	**100 µM Mino % wound closure of starting distance**	**paired student’s t-test**
**24 h**	79.7 ± 6.6	87.5 ± 5.1	* n=17, p=0.0029
**48 h**	47.5 ± 21.6	59.0 ± 9.6	n=17, p=0.0563
**72 h**	26.5 ± 22.1	35.8 ± 16.6	* n=14, p=0.0305

All values are given as mean ± SD. Minocycline supplement (Mino), number of samples (n), error level (p), * statistical significance p<0.05.

Analysis of the controls vs. +Mino revealed a significant difference in wound closure after 24 h and 72 h. Minocycline delayed the wound closure in primary glial cell cultures compared to control cells. In the 48 h experiment, this delay was also evident but not significant. This minocycline-mediated delay in wound closure is illustrated by phase contrast images in Figure 9 B.

#### Western Blot Analysis

**Figure 10:**
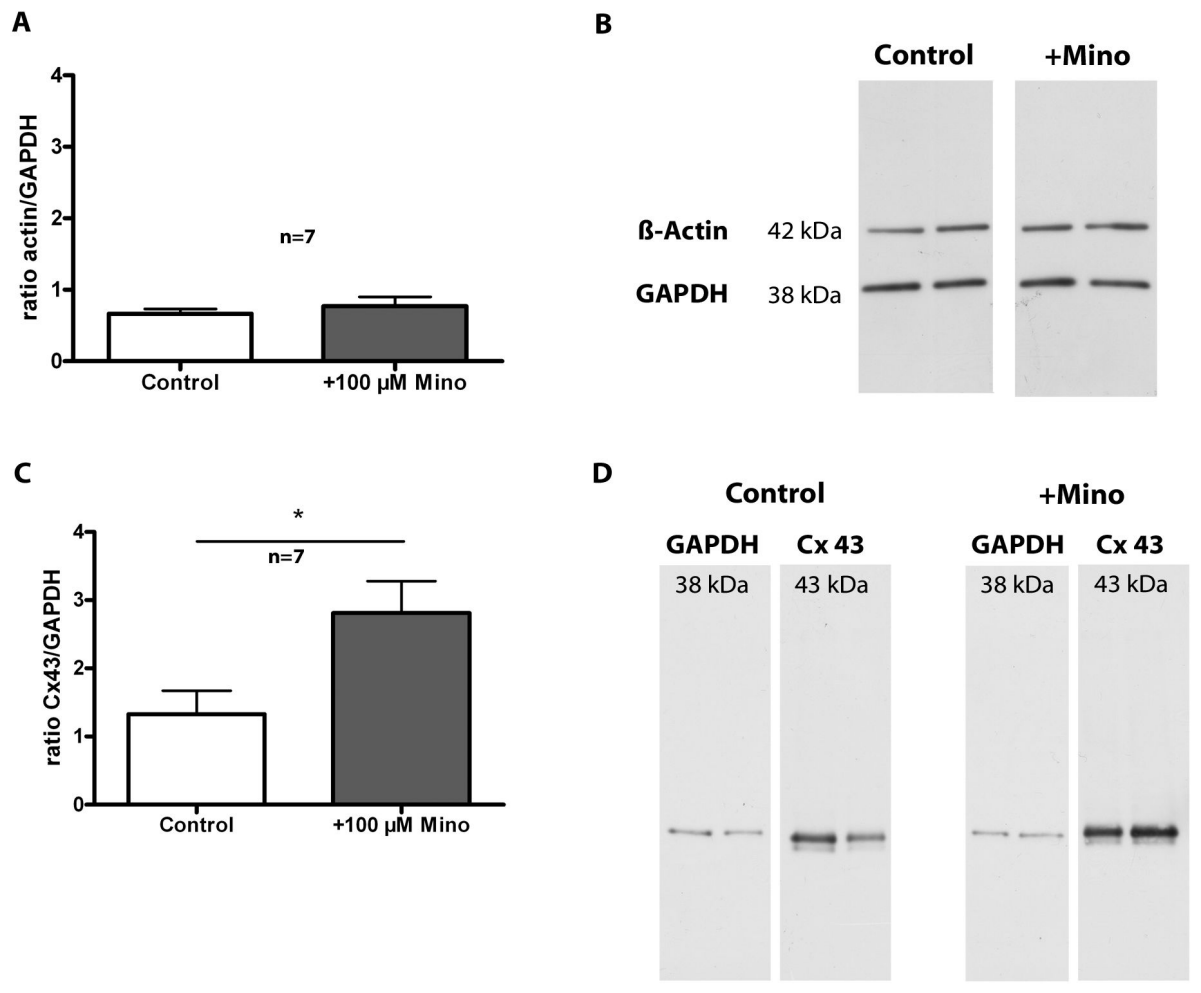
Western Blot analysis of primary glial cell cultures. Primary glial cell cultures were incubated for 72 h with 100 µM minocycline (+Mino). Control cells received medium without any supplement. Values in (A) are given as mean ratio of β-actin/GAPDH density ± SD and values in (C) are given as mean ratio of Cx43/GAPDH density ± SD. (**B**) shows representative images of β-actin bands of control and +Mino cells with corresponding GAPDH bands. (**D**) displays representative images of Cx43 bands of control and +Mino cells with corresponding GAPDH bands. Minocycline did not change the expression of β-actin but enhanced the expression of Cx43 compared to control cells. Statistical analysis was done with a paired Student’s t-test. A p-value ≤ 0.05 was considered to be statistically significant and is marked with *. The number of samples was n = 7.

To further explore the changed by minocycline migration behavior of primary glial cells, the amount of Cx43 and β-actin in these cultures was analyzed. These proteins are known to play a role in migration. β-actin is a cytoskeletal protein and Cx43 is a hemichannel protein found in gap junctions that are localized between cells. Minocycline incubation for 72 h had no effect on β-actin expression if compared to control cells. Figure 10 A shows the quantification of β-actin expression as the ratio of β-actin density/GAPDH density. Control cells revealed a ratio of 0.7 ± 0.2 and +Mino cells 0.8 ± 0.3 (p=0.7285). Representative bands of β-actin and the corresponding GAPDH signal are shown in Figure 10 B. The quantification of Cx43 signal is provided in the diagram in Figure 10 C. Incubation with 100 µM minocycline for 72 h significantly increased the protein expression of Cx43 compared to control cultures. Control cells revealed a ratio of 1.3 ± 1.0 and +Mino cells 2.8 ± 1.2 (p=0.0445). Representative images of the bands are provided in Figure 10 D.

## Discussion

The major findings of our study are as following: already a low concentration of 10 µM minocycline showed a neurotoxic effect on motor neuron survival. However this effect was mild in comparison with the effect of high minocycline concentration and was found only with prolonged minocycline incubation time. This low concentration did not affect microglia and astroglia activation. A high concentration of 100 µM minocycline showed enhanced effects: it reduced motor neuronal survival, neuronal survival in general and the percentage area of DAPI-stained cell nuclei in organotypic spinal cord cultures. Treatment with high-dosed minocycline inhibited microglia activation highly. Moreover, 100 µM minocycline impaired the formation of an astroglial cover on the surfaces of the organotypic spinal cord slice cultures and co-cultures. Improvement in the regenerative environment by the reconstruction of the ventral root did not rescue motor neuron survival and astroglia from the minocycline-induced effects. Treatment with 100 µM minocycline revealed mild toxic effects on dispersed astroglial cultures and impaired glial migration.

Neonatal motor neurons, similar to the ones studied in our organotypic spinal cord cultures, are dependent on target contact and die after axotomy [59–62]. To overcome this problem, we added GDNF to our cultures [42]. Control cultures showed good motor neuronal survival. Nevertheless, minocycline induced motor neuron cell death even with a low concentration of 10 µM minocycline treatment starting at DIV 1 in culture. The neurotoxic effect which we found is in contrast to other previous cell culture studies that have described neuroprotective effects at even higher minocycline concentrations [28,29,34]. For example, Guo and colleagues [31] treated cells of a motor neuron cell line with 25 µM minocycline and observed a neuroprotective effect against hypoxic cell death. Pi et al. [30] added 10-100 µM minocycline to cerebellar granule neurons and found a dose-dependent neuroprotective effect against glutamate excitotoxicity. The neurotoxic/neuroprotective effects of minocycline may be dependent on the paradigm and the complexity of the cell culture system employed.

The neurotoxic effects in our organotypic spinal cord cultures were accelerated with a high minocycline concentration of 100 µM. Interestingly the neurotoxic effect was accompanied by an enhancement in neurite staining, although the corresponding cell somata were missing. One property of minocycline is its inhibitory effect on microglia activation [22,63,64] which we were also able to demonstrate in our organotypic spinal cord cultures. If this enhanced neurite staining is due to a toxic effect of minocycline on microglia or an effect of suppression of the activation of microglia, future experiments have to show. Keilhoff et al. [37] found that minocycline reduced the phagocytic activity of Schwann cells. Keeping this in mind, we speculate that the loss of microglial action causes a delayed debris clearance and the remaining fibers can still be stained with anti-pan-NF antibody in the minocycline-treated cultures. Other reasons could be that minocycline induced a change in regulation or transport of the neurofilaments. Future experiments have to look for this in more detail.

In addition to pro-inflammatory functions, microglia activation also exhibits beneficial effects on injured tissue. Microglia which phagocyte cell debris reduce the amount of growth inhibitory molecules (such as the myelin components Nogo A and myelin-associated glycoprotein) [57,65,66]. Moreover, microglia has been shown to protect neurons from nitric oxide toxicity [67] and ischemia-induced cell death [68]. Inhibition of these supportive functions could be another reason for the neuronal death observed in our model.

An additional stressor was found after analyzing the effect of 100 µM minocycline on astroglia. High-dosed minocycline hampered the motility of astrocytes which could result in the prevention of the formation of the glia cover on the surface of the spinal cord slices. In spinal cord injury, the injury is followed by scar tissue formation, which serves as a functional barrier to nerve fiber regeneration. However, a beneficial role of scar tissue formation, particularly during the acute phase of spinal cord injury, has also been described. For example, Cui et al. [69] showed that the removal of astrocytes after central nervous system (CNS) injury resulted in a decrease of glutamate transporter expression. Glutamate transporters, however, are required to remove glutamate from synaptic clefts. The lack of glutamate transporters results in excitotoxic levels of glutamate and subsequent neuronal death. Moreover, Chen and colleagues [70] reported neuroprotection from nitric oxide toxicity by astrocytes via a glutathione-dependent mechanism. Furthermore, astrocytes provide trophic support after CNS injury [9,71]. Bush et al. [72] described a role for astrocytes in regulating the immune response, repair of the blood–brain-barrier and neuronal survival and outgrowth. The removal of these beneficial effects from reactive astrocytes by the inhibition of astroglial motility and subsequently the formation of the glia cover on the slice surface in the minocycline-treated cultures could have been an additional factor that caused motor neuron cell death in our cultures. Motor neurons require trophic support and the scavenging activity of glia cells. Thus, the absence of the fine-tuned glial orchestra may amplify the minocycline-induced death of motor neurons in the organotypic spinal cord cultures.

To more closely examine the effect of 100 µM minocycline on astroglia, we analyzed primary glial cell cultures after 72 h of minocycline treatment. Consistent with previous studies [73] (osteoblasts) [74], (human aortic smooth muscle cells) [75], (osteoblastic bone marrow cells), we found a mild toxic effect on cells incubated up to 7 days with minocycline. This effect may explain the reduction in the astroglial percentage stained area observed in cultures treated at DIV 4 with minocycline.

Minocycline also interfered with glial cell migration in a scratch assay. Wound healing was slower in cell cultures treated with minocycline compared to controls. Due to the toxic effect shown in the MTT analysis, the delayed wound healing could also be due to a loss of cells in the minocycline treated group. Against this is that we detected a delayed wound healing already after 24 h; a time point at which we could not detect a toxic effect in the MTT. The reduced migration ability of astroglia caused by minocycline could explain the impaired surface-glia-cover formation observed in the organotypic cultures. Minocycline prevented astroglia from migrating from the spinal cord tissue to the surface of the slice. Several proteins have also been suggested to play a role in cell migration and motility, which may be targets of minocycline activity. One important factor for glial migration is Cx43. Connexins are proteins that cluster to form hemi-channels or connections in the cell membrane. 2 hemi-channels from neighboring cells can form a gap junction, which connects the cells’ cytosols [76]. Gap junctions show various channel-dependent and channel-independent functions, which are important for CNS homeostasis, cell migration and cell motility [77]. Cx43 is the most expressed connexin in astroglia and is involved in the formation of the astroglial syncytium in the CNS [78]. Because of its important role in migration, we examined the expression of Cx43 in primary glial cell cultures after 72 h of incubation with 100 µM minocycline. Minocycline treatment increased the protein expression of Cx43 in our cell cultures. Several studies have previously described an inhibitory effect of activated microglia on astroglia coupling [77,79,80]. Retamal and colleagues [81] found that activated microglia regulate the Cx43 hemi-channels and gap junctions of astroglia via pro-inflammatory cytokines. Pro-inflammatory cytokines decreased the levels of Cx43 and increased membrane permeability. Minocycline inhibits the activation of microglia, which was shown in our study, and has been well described in the literature for its anti-inflammatory effect [21–23]. The primary glia cell cultures used for our western blotting studies also contained some microglia. In primary cell cultures, a low microglia activation state is expected because there are always cells dying, and cell debris is removed. If these microglia are now inhibited by minocycline, then a higher level of Cx43 in the +Mino cultures with inhibited microglia activation is expected compared to control cells, which are permanently influenced by low microglia activation.

There have been conflicting results in the literature regarding the effect of high/low Cx43 levels on cell migration. Most studies have examined the effects of a reduction in Cx43. McDonough et al. [82] found that reduced gap junction formation enhanced the migration of glioma cells in vitro, whereas [83] showed that depletion of Cx43 in astroglia delayed wound closure and decreased cell proliferation, indicating a direct impairment of the migration of transfected astrocytes. Our study is consistent with McDonough’s study [82]. In our study, control astroglial cultures with a lower Cx43 expression level showed a higher rate of migration compared to minocycline-treated cultures with higher levels of Cx43. Conveying this to our organotypic cultures, the enhanced coupling between the astroglia resulted in a loss of migratory function and prevented the formation of the glia cover. Additionally, there is an ongoing debate in literature about adverse effects of high Cx43 levels after spinal cord injury [84–86]. It seems that neurodestructive events can be enhanced by high Cx43 levels because toxic molecules released by dying cells can be spread via the astrocytic network resulting in high Ca2+ entry in neurons and thus inducing neuronal death.

In addition to the spinal cord cultures, we used spinal cord co-cultures with a peripheral nerve graft to reconstruct the ventral root. Motor neuron survival and axonal outgrowth after root avulsion appeared to be supported if a nerve graft was immediately added. Schwann cells of the nerve graft secrete neurotrophic factors and offer a scaffold for regenerating axons to grow into [38]. Motor neurons suffering from a proximal injury, similar to the injury induced in this study, show enhanced death compared to more distal injuries [54,55]. Gu et al. [54] showed an improvement in the regeneration and survival of motor neurons in adult rats after reimplantation of the ventral root in a root avulsion model. However, the functional recovery was not convincing because some animals revealed positive electromyographies but lacked behavioral recovery. Moreover, patients with an intraspinal brachial plexus injury showed improvement in sensory and motor functions with the repair of the spinal root [87]. In addition, a peripheral nerve graft serves as a source of Schwann cells in our model. Schwann cell transplantation has been shown to promote axonal regeneration in spinal cord injury by creating a permissive environment enriched in neurotrophins [56–58]. Ianotti and colleagues [88] demonstrated that the combination of GDNF treatment and Schwann cells enhanced axonal regeneration and remyelination more than each of the factors alone. For this reason, we investigated whether minocycline treatment on spinal cord co-cultures affected neuronal survival in a similar manner to spinal cord cultures without a peripheral nerve graft. An improved survival environment for motor neurons due to the nerve grafting was not obvious. A comparison of the organotypic control cultures with and without the reconstructed ventral roots revealed no differences in the number of surviving motor neurons (data not shown), indicating that survival due to the GDNF supplement overlaps with the expected effect. However, in the organotypic co-cultures minocycline treatment reduced the number of neurons and increased the percentage of neurite staining with anti-pan-NF. Similar to our results obtained from the organotypic cultures without a peripheral nerve graft, the glia cover formation was inhibited in early/long-term minocycline treated co-cultures but did not affect late/short-term treated co-cultures. Thus, promotion of the regenerative environment by Schwann cells was unable to rescue motor neuron cell death and did not compensate for the minocycline effects on astroglia.

However, it seems that the neuroprotective effects of minocycline may be dependent on the paradigm and the complexity of the cell culture system employed. In comparison with disperse cell cultures organotypic cultures have several advantages for drug screening. Organotypic cultures maintain their three-dimensional tissue organization in culture [45]. They contain all cell types and maintain their cell-cell-interactions, thereby modeling *in vivo* conditions to a higher degree compared to monolayer cell cultures. The minocycline concentrations used in this study are consistent with previous studies. Nevertheless, organotypic cultures are of course unable to completely model *in vivo* situations and diseases. Therefore, they only can provide the first idea what minocycline can do in *vivo*. Conflicting effects were also demonstrated in several cell culture studies, which used concentrations that varied between 10 and 100 µM. Moreover, Pi et al. [30] showed a dose-dependent reduction in glutamate toxicity on primary neurons that were pre-treated with minocycline. Treatment with 100 µM minocycline has been shown to prevent the formation of the mitochondrial permeability transition pore [27] and to affect mitochondrial function [28,36]. Most *in vivo* studies use approximately 40-90 mg/kg minocycline. Studies have revealed both neuroprotective effects of minocycline as well as negative effects using similar concentrations. For example, Lee et al. [18] used a starting dose of 90 mg/kg and an ongoing dose of 45 mg/kg in rats with a mild contusion spinal cord injury. Rats showed a reduced lesion size and fewer apoptosis markers compared to controls. Cho and colleagues [19] reported the facilitation of motor recovery and attenuation of mechanical hyperalgesia with an injection of 40 mg/kg minocycline after a hemisection in rat spinal cords. A concentration of 45 mg/kg or 60 mg/kg was used by [32] and induced enhanced MPTP toxicity in dopaminergic neurons.

However, the first results of a clinical trial with minocycline for the treatment of spinal cord injury were recently published [89]. Casha and colleagues monitored the serum levels of minocycline in patients to improve the oral minocycline dose and result in comparable serum levels similar to those used in animal studies. For this reason, they administered a high starting dose of 800 mg with an ongoing dose of 400 mg minocycline and found a significant improvement in motor score in patients with cervical injuries. The comparison between concentrations used *in vitro* and *in vivo* is difficult because of the distinct features of the systems. Though, *in vivo* and *in vitro* studies may not be easily used to determine the optimal drug concentration for human application; however, these studies may provide clues regarding the beneficial functions or toxicity of minocycline treatment.

## Conclusion

Minocycline is an antibiotic drug that has been described to have neuroprotective, anti-inflammatory and anti-apoptotic effects in various models of neurodegenerative diseases. However, conflicting results have been obtained by a number of different studies. In our model of motor neuronal survival and regeneration, minocycline of 10 and 100 µM failed to reveal neuroprotective effects, but we reproduced its inhibitory effect on microglia activation. Thus, minocycline demonstrated anti-inflammatory function and prevented beneficial microglia activity which is necessary for neuroprotection/-regeneration.

Moreover, minocycline impaired astroglia migration, increased Cx43 protein expression and prevented the formation of the glia cover on the surface of our organotypic spinal cord slices. We speculate that minocycline also could reduce glia scar formation *in vivo*. Additionally, Cx43 might be a possible molecular target of minocycline. During the early phase of CNS injury, reactive astroglia, which are the major component of the glia scar, appear to have a beneficial effect in regulating the immune response and glutamate balance.

Impairment of the functions of both glia types may be one factor being co-responsible for the lack of neuroprotection and contributing to the neurodegenerative effects of minocycline. Improvement in the motor neuron environment after injury by the addition of a peripheral nerve graft with Schwann cells did not improve the outcome of our cultures.

Taken together, early minocycline administration *in vivo* after spinal cord injury should be reconsidered because of the inhibition of beneficial glia functions during the acute phase of the injury. Administration during the chronic phase with a fully established glia scar may be more promising since minocycline reduced the glia cover in our cultures even after glial formation. Late/short administration of minocycline may also reduce the inhibitory effects of the glia scar on regrowth in the chronic injury phase.
